# An Evaluation of Pretrained Generative Models for Augmenting Small Health Data: Comparative Modeling Study

**DOI:** 10.2196/88678

**Published:** 2026-06-15

**Authors:** Margerie Huet-Dastarac, Fida K Dankar, Dan Liu, Samer El Kababji, Lisa Pilgram, Khaled El Emam

**Affiliations:** 1 School of Epidemiology and Public Health, Faculty of Medicine, University of Ottawa, 451 Smyth Rd, Ottawa, ON, K1H 8M5, Canada, 1 613-562-5800; 2 Research Institute, Children's Hospital of Eastern Ontario, Ottawa, ON, Canada; 3 Department of Nephrology and Medical Intensive Care, Charité - Universitaetsmedizin Berlin, Berlin, Germany

**Keywords:** binary classification, machine learning, data augmentation, synthetic data generation, tabular data, small data regime

## Abstract

**Background:**

Synthetic data generation (SDG) has emerged as a promising solution to address data scarcity in health care, where privacy concerns, regulatory barriers, and the high cost of data acquisition limit access to real patient datasets. Machine learning models in this domain often operate in low-data regimes, with training set sizes as low as 20 and a median dataset size of around 600 records—conditions that hinder model generalization and increase the risks of overfitting and bias. SDG addresses these challenges by producing artificial samples that mimic real-world patient data, enabling robust and privacy-preserving model development.

**Objective:**

This study was a comprehensive assessment of SDG-augmented training across a wide array of models—both pretrained and non-pretrained—for outcome prediction in 13 health care datasets. For small datasets of sizes 50 and 350 records, we answer 3 key questions: (1) Do pretrained SDG models generate more effective augmentations than their non-pretrained counterparts for small datasets? (2) Is augmentation beneficial for both pretrained and non-pretrained classifiers for small datasets? (3) Among 3 state-of-the-art classification models, which offers the best predictive performance on small datasets? The workload that this study aimed to improve was binary classification.

**Methods:**

The 3 classifiers considered were light gradient boosting trees, large language models (LLMs) adapted to tabular data, and Tabular Prior-Data Fitted Network (TabPFN), a transformer-based method that has become the new state of the art in terms of tabular data classification. Each classifier was augmented through different SDG methods: current state-of-the-art techniques (Bayesian networks, conditional tabular generative adversarial networks, tabular variational autoencoders, and sequential trees) and the use of LLMs for tabular data generation.

**Results:**

Augmented TabPFN demonstrated superior performance, yielding significantly higher area under the curve and integrated calibration index scores compared to other classifiers. Post hoc analysis revealed that, for the dataset sizes examined, SDG and LLM models exhibited overfitting tendencies. Notably, simple dataset augmentation through sampling with replacement achieved performance comparable to that of SDG-based and LLM-based augmentation methods for TabPFN, suggesting that gains were primarily driven by increased sample size rather than SDG.

**Conclusions:**

Given its strong performance and minimal computational overhead, we recommend augmenting TabPFN through sampling with replacement as the optimal approach for small-data binary classification tasks. This method achieves performance comparable to that of more complex SDG techniques while offering substantial computational advantages.

## Introduction

### Background and Study Objectives

Machine learning predictive modeling applications in health care often suffer from limited access to real patient data due to privacy concerns, regulatory constraints, and the high cost of data acquisition. Recent reviews have identified that most machine learning studies rely on training models on datasets of insufficient sizes [1-5]. This shortage in data availability—referred to as a low-data regime—introduces challenges such as overfitting [1,6], biased learning, and reduced model robustness [2].

Synthetic data generation (SDG) has been proposed as a potential solution by augmenting training datasets with artificially generated samples that closely mirror real patient data. Increasing the size of the training dataset is generally associated with improved predictive performance in machine learning models [7]. However, it remains unclear whether the benefits of augmentation arise from the fidelity of synthetic samples or simply from the increased sample size. In addition, augmentation can be interpreted as a form of regularization, where synthetic examples increase the diversity of the training data by generating additional variations from the same underlying population [8].

A key question is, therefore, whether pretrained SDG models, when used for prediction and augmentation, can perform better than non-pretrained models and improve the performance of predictive clinical classification tasks. This study presents a large-scale evaluation of the impact of data augmentation by SDG on both pretrained and non-pretrained prediction models. We consider 2 realistic low-data regime scenarios for health datasets: 50 and 350 records. This definition of a low-data regime is consistent with current practices, where the median size of the training datasets used in clinical prediction tasks can be as low as 20, with a median value around 600 records [3,9].

We structured our study to answer the following specific questions in the case of a low-data regime:

- Q1. Is data augmentation using pretrained SDG models outperforming data augmented by non-pretrained SDG models?
- Q2. What is the effect of augmentation on pretrained and non-pretrained classification models?
- Q3. What is the best clinical predictive classification model among gradient-boosted trees, large language models (LLMs), and Tabular Prior-Data Fitted Network (TabPFN)?

We challenge the assumption that augmentation through SDG is necessary for improving clinical prediction in low-data regimes by systematically comparing SDG, LLM-based augmentation, and resampling methods. We demonstrate that the gains are driven primarily by increased sample size, with simple sampling with replacement achieving performance comparable to or exceeding that of complex generative approaches.

### Previous Work

For nontabular data, data augmentation has been applied to address the low-data regime problem, such as in imaging, video, and natural language processing data [10-15], and it has been shown to be a viable solution to address the problem of incomplete and unbalanced time series datasets [16-19]. However, there has been limited work on the evaluation of data augmentation in the context of tabular health data for clinical predictive workloads.

Commonly used SDG models are conditional tabular generative adversarial networks (CTGANs) [20], tabular variational autoencoders (TVAEs) [21], Bayesian normalization methods [22-25], and sequential decision trees [26-29]. However, classification models are not the only ones affected by the small dataset size available. In fact, the SDG models themselves may experience overfitting when trained on small datasets, raising questions about the quality of synthetic samples under the low-data regime.

Recent work has highlighted the potential of pretrained transformer models, such as LLMs, for tasks involving tabular data, specifically SDG and classification [30,31]. LLMs have been adapted from their original textual domain to tabular data through methods that account for dataset-specific properties, such as column-order and row invariance. Fine-tuning such models enables their application not only to classification but also to SDG [32]. Several methods—including Curated LLM (CLLM) [33], LLMOverTab [34], and Pred-LLM [35]—explicitly leverage LLM pretraining on large datasets for tabular data generation in low-data regimes.

However, recent work has noted that LLMs may not yet perform classification at a level comparable to traditional machine learning models, underscoring the importance of systematically evaluating LLMs in clinical contexts [36]. Recent models such as the TabPFN were designed for classification and represent a transformer architecture pretrained on synthetic tabular data. While TabPFN demonstrates promising performance on datasets ranging from 650 to 10,000 records [37], this size range fails to address the reality of clinical prediction tasks, where datasets commonly contain fewer than 600 records [3,9]. This gap is particularly significant given that many clinical prediction tasks must operate in low-data regimes due to data privacy constraints and the rarity of certain conditions.

## Methods

The workload that this study aimed to improve was predictive binary classification.

### Ethical Considerations

This project was approved by the Research Ethics Board of the Children’s Hospital of Eastern Ontario Research Institute, protocol 24/80x. Because the datasets used in this study were deidentified, obtaining participant consent was waived by the Research Ethics Board of the Children’s Hospital of Eastern Ontario Research Institute. This project adhered to the Declaration of Helsinki.

### Models Evaluated

The pretrained and non-pretrained models used in this study are summarized in Table 1. We use the term *non-pretrained* to denote models trained directly from scratch on the available data, in contrast to LLMs and TabPFN, which rely on extensive pretraining on real and synthetic data.

**Table 1.** Overview of the models evaluated in this study.

Purpose	Non-pretrained models	Pretrained models
Classification model	LGBM^a^	DistilGPT2 Llama 1B Llama 8B fine-tuned on UltraMedical dataset TabPFN^b^ version 2
Synthetic data generation (generative models)	Sequential decision trees Bayesian network CTGAN^c^ TVAE^d^	DistilGPT2 Llama 1B Llama 8B fine-tuned on UltraMedical dataset

a LGBM: light gradient boosting machine.

b TabPFN: Tabular Prior-Data Fitted Network.

c CTGAN: conditional tabular generative adversarial network.

d TVAE: tabular variational autoencoder.

### Non-Pretrained Generative Models

We used 4 commonly applied generative modeling methods to generate new observations for structured tabular data. CTGAN is a conditional generative adversarial network specifically adapted for tabular data, which captures complex feature distributions through adversarial training [38-40]. TVAE uses variational autoencoding to model the joint distribution of tabular features, enabling flexible data synthesis [21,41,42]. Bayesian networks represent probabilistic relationships between variables through directed acyclic graphs, allowing for the generation of synthetic data consistent with estimated dependencies [22-25]. Sequential trees generate synthetic data by recursively partitioning the feature space in a manner similar to decision trees, ensuring that complex conditional dependencies are preserved [26-29]. All 4 approaches have been widely adopted in recent work on tabular data synthesis.

Categorical and continuous features were identified based on dataset metadata. Continuous variables were normalized using training-set statistics, and categorical variables were one-hot encoded where required for the modeling task. Missing values were handled using the default mechanisms of each model (eg, a missing categorical value was treated as a valid category in the modeling). These are described in the documentation or the implementation of the generative models. Hyperparameters followed standard recommended settings as described in the original implementations. Synthetic samples were generated by unconditional sampling from the fitted models and then inverse-transformed back to the original feature space.

Sequential synthesis was implemented using Aetion Generate, a commercial product from Aetion, and the last 3 methods were implemented using an open-source Python package, Synthcity [43]. The *pysdg* library [44], our publicly available adaptation of Synthcity, provides further preprocessing and postprocessing on top of Synthcity.

### Pretrained Generative Models

Fine-tuning an LLM involves adapting a pretrained model to a specific task or domain by training it on a smaller, task-specific dataset such as processing tabular data instead of free text. Fine-tuning leverages the general knowledge already encoded in the model from pretraining on vast amounts of data, allowing the model to specialize without requiring training from scratch. During fine-tuning, the model’s parameters are updated to align its outputs with the desired behavior. We used the low-rank adaptation (LoRA) approach [45], which is commonly used for efficient fine-tuning.

LLMs are pretrained on large-scale text corpora and are therefore designed to process textual input and generate textual output. Several strategies have recently been proposed to adapt LLMs for tabular learning tasks. In this study, we used the PredLLM framework, which reformulates each tabular record into a natural language sentence following the pattern “*column name is value,...*” for fine-tuning the LLM. To prevent the model from exploiting column order as information, the input columns were randomly shuffled during training. Finally, the target variable was consistently placed at the end of the sequence to ensure that predictions incorporated information from all other features. The variables of a record are therefore generated column by column, completing with the outcome variable.

Relying on fine-tuning on serialized tabular training data, the LLMs were used for SDG without an explicit system prompt. Each synthetic record was generated by conditioning the model on a randomly selected feature-value pair sampled from the empirical distribution of the training data. Given this partial input, the model autoregressively completed the remaining features in a fixed predefined order learned during fine-tuning. Generated outputs were parsed back into tabular form using the known schema. More details are provided in Section B in Multimedia Appendix 1.

As described in Table B2 in Multimedia Appendix 1, we used 3 LLMs of various sizes: DistilGPT2 and Llama 1B, pretrained on general data, and Llama 8B, specifically pretrained on the UltraMedical dataset [46], which contains more than 400,000 samples of synthetic and manually curated biomedical instructions.

### Non-Pretrained Classification Models

In this study, the chosen classification non-pretrained model was a light gradient boosting machine (LGBM) [47]. Tree-based models are the most common type of machine learning prediction methods used in clinical research [3]. They perform better than linear models, such as logistic regression [48-52], and they were found to perform better than deep learning models on tabular datasets [53,54].

Model tuning used 5-fold cross-validation and Bayesian optimization [55]. The range for the tuning parameters was previously suggested [56-59], and these are summarized in Multimedia Appendix 1. High-cardinality variables were converted to embeddings [60] using a scheme similar to target encoding.

### Pretrained Classification Models

The same pretrained models used for the generation of data were also used for classification. Their application to classification tasks can be seen as only the last step of the generation process, where the outcome variable is generated for a record based on all previous variables.

Another model, TabPFN, is a transformer-based model designed to perform classification and regression tasks on tabular data. It is trained on a large corpus of synthetic classification tasks with known Bayesian-optimal solutions, from which the model learns to approximate posterior class probabilities directly from features and labels without requiring further training on new tasks. This allows it to generalize effectively across diverse tabular datasets and make predictions quickly, particularly excelling in low-data regimes. Unlike traditional machine learning models that rely on iterative training and hyperparameter tuning, TabPFN offers fast, zero-shot inference through an in-context learning mechanism.

### Research Questions

#### RQ1: Is Data Augmentation Generated by Pretrained SDG Models Outperforming Data Augmented by Non-Pretrained SDG Models?

We trained 2 classifiers with augmented datasets: LGBM and TabPFN. The synthetic samples of the augmented data were generated by the 3 pretrained SDG approaches based on different LLMs and 4 non-pretrained models.

The 2 classifiers were trained for binary classification tasks. We assessed the downstream utility by reporting the area under the curve (AUC) score, integrated calibration index (ICI), and the corresponding n′ (the number of synthetic samples).

We performed 1-tailed paired permutation tests on both the AUC and ICI metrics across the datasets to comprehensively evaluate whether pretrained SDG models outperform non-pretrained SDG models. A 1-tailed permutation test was chosen because our hypothesis was directional—namely, that pretrained SDG models would outperform non-pretrained SDG models under the small data regime by leveraging knowledge from their pretraining.

We selected the models yielding the highest AUC and the ones yielding the lowest ICI in each category (pretrained and non-pretrained) for each dataset and performed the permutation tests. Testing AUC allows us to determine if one model demonstrates significantly better discrimination—the ability to correctly rank outcomes—whereas testing ICI evaluates whether one model provides more accurate probability estimates through improved calibration.

#### RQ2: What Is the Effect of Augmentation on Pretrained and Non-Pretrained Classification Models?

This question entails a comparison of whether to use data augmentation or not for pretrained and non-pretrained classifiers. We considered TabPFN as a pretrained classifier and LGBM as a non-pretrained classifier. We used the most beneficial data augmentation method, selected through the analysis answering the first question of this study (RQ1), and compared the classification results to the no augmentation baselines.

These 2 classifiers were trained on the same prediction tasks as RQ1, and the same downstream utility metrics were reported. We performed 1-tailed paired permutation statistical tests. One-tailed permutation tests were chosen because our hypothesis was directional—namely, that augmentation would improve classification performance for models under the small data regime.

#### RQ3: What Is the Best Clinical Prediction Classification Model Among LGBM, LLMs, and TabPFN?

To answer this final question, we compared the performance of each of the considered classifiers—LGBM, fine-tuned LLMs, and TabPFN—each in their best-performing augmentation configuration, as determined by answers to questions RQ1 and RQ2. Six 1-tailed permutation tests were performed for each low dataset regime: 3 on AUC metric and 3 on ICI—LGBM versus LLM, TabPFN versus LGBM, and TabPFN versus LLM.

For each dataset and the 2 low-data regimes, we reported in Multimedia Appendix 1 the best-performing methods in terms of aggregated AUC, corresponding ICI, and n′.

### Study Design

To address our research questions, we designed a comprehensive evaluation procedure. We summarize the main tasks below and expand on some of the key ones after that.

#### Evaluation Scope

We evaluated 3 types of binary classifiers (LGBM; LLMs—DistilGPT2, Llama 1B, and Llama 8B; and TabPFN) on binary classification tasks with different augmentation strategies. An important limitation was that LLM classifiers were only evaluated without augmentation due to computational constraints. Fine-tuning LLMs with different quantities of synthetic samples would require 360 days using 1 NVIDIA-RTX-A6000 GPU 48 GB RAM (NVIDIA Corporation). This computational intensity makes such extensive fine-tuning impractical for the intended end users. Therefore, LLMs were used only as baseline classifiers and as SDG models. A detailed analysis of computational requirements is provided in Section D in Multimedia Appendix 1.

#### Dataset Preparation

We used 13 clinical datasets (summarized in Table 2 and further detailed in Section E in Multimedia Appendix 1). For each dataset, we simulated 2 low-data regimes by randomly sampling subsets of n_0_=50 and n_0_=350 records. We created hold-out validation and test sets of 10,000 records each from the remaining data, which are fixed to evaluate the different models fairly. We used stratified sampling for these sets to keep the original prevalence of target classes.

**Table 2.** Summary of the 13 large real-world datasets from which 50 and 350 records are sampled, training synthetic data generation and classification models, and 10,000 records are sampled as test sets.

Dataset name	Description	Number of variables
COVID (COVID-19)	A dataset that covers COVID-19 health records of Canadians collected by Esri Canada	7
Canadian Community Health Survey (CCHS)	A pooled version of survey data across multiple years that gathers health information for the Canadian population	8
COVID Survival (Nexoid)	A secondary web-based survey dataset concerning COVID-19 survival prediction collected by the Nexoid company in London, UK	19
FDA Adverse Event Reporting System (FAERS)	A database that contains adverse events and medication error reports submitted to the FDA^a^	7
Texas Inpatient Data (Texas)	A dataset on discharges from Texas hospitals	11
Washington State Hospital Discharge (Washington)	A dataset that collects the hospital discharge information from the HCUP^b^ state inpatient database for 2007	8
Basic Stand Alone Inpatient Claims (BSA)	A dataset that contains the claim-level information from 2008 Medicare inpatient claims	6
Washington State Hospital Discharge (Washington 2008)	A dataset that collects the hospital discharge information from the HCUP state inpatient database for 2008	18
California Hospital Discharge (California)	A dataset that collects the hospital discharge information from the HCUP state inpatient database for 2007	16
Florida Hospital Discharge (Florida)	A dataset that collects the hospital discharge information from the HCUP state inpatient database for 2007	12
New York Hospital Discharge (New York)	A dataset that collects the hospital discharge information from the HCUP state inpatient database for 2007	14
Medical Information Mart for Intensive Care III (MIMIC-III)	A dataset that comprises deidentified health data associated with intensive care unit admissions	13
Better Outcomes Registry & Network (BORN)	A dataset that collects data about pregnancy, birth, and childhood in the province	20

a FDA: Food and Drug Administration.

b HCUP: Healthcare Cost and Utilization Project.

#### Synthetic Data Generation

We used 7 SDG models: 3 pretrained models (DistilGPT2, Llama 1B, and Llama 8B) and 4 non-pretrained models (sequential decision trees, Bayesian network, CTGAN, and TVAE). For each SDG model and each dataset, we generated synthetic samples in varying quantities (n′) ranging from 5 to 10,000 records, following a geometric series (details provided in the Augmentation Scheme section). For each n′, we generated 5 synthetic datasets to account for model stochasticity and averaged the performance results across the augmented datasets.

#### Data Augmentation

For each combination of real data subset (n_0_) and synthetic data (n′), we created augmented datasets by concatenating the real and synthetic data. This process was repeated for all SDG models and both low-data regimes (n_0_=50 and n_0_=350).

#### Model Training and Evaluation

For each classifier (except LLMs), we trained models on original data only (no augmentation), augmented data from pretrained SDG models, and augmented data from non-pretrained SDG models.

We evaluated each model’s performance using the hold-out validation set, calculating AUC and ICI. For augmented datasets, we averaged the performance across the 5 synthetic datasets for each n′. Therefore, for each n′ value, we trained 5 models.

#### Optimal Augmentation Selection

For each dataset, classifier, and SDG model combination, we identified the optimal n′ that yielded the best AUC on the validation set. We recorded the corresponding AUC, ICI, and n′ for further analysis.

#### Statistical Analysis

To answer RQ1, we performed 1-tailed paired permutation tests comparing the best-performing pretrained and non-pretrained SDG models for both AUC and ICI metrics. For RQ2, we conducted 1-tailed paired permutation tests to compare augmented versus nonaugmented performance for LGBM and TabPFN for both AUC and ICI metrics. To address RQ3, we performed six 1-tailed permutation tests (3 each for AUC and ICI) comparing LGBM, LLMs, and TabPFN in their best-performing configurations.

#### Reporting Results

We summarized the results in tables and figures, showing the performance metrics (AUC and ICI) and optimal n′ for each combination of classifier, augmentation strategy, and dataset. We reported the outcomes of all statistical tests, indicating the magnitude and significance of the differences between methods.

This overall evaluation design allows us to systematically assess the effectiveness of data augmentation techniques, compare pretrained and non-pretrained models, and identify the best-performing clinical prediction classification models under low-data regimes, while acknowledging and accounting for practical computational constraints.

### Augmentation Scheme

Augmented data represents the concatenation of the original data (size n_0_) and the synthetic data (size n′). To assess performance under varying low-data regimes, we simulate 2 levels of data scarcity by randomly sampling a subset of n_0_=50 and n_0_=350 records from each of the 13 datasets (see Table 2 for a summary). For each dataset, we identify the optimal number of synthetic samples (n′) and determine the best-performing SDG method. Unlike prior studies, which often fix an arbitrary number of synthetic samples across datasets, our approach adapts the number of augmented examples based on dataset-specific performance criteria.

**Figure 1.**
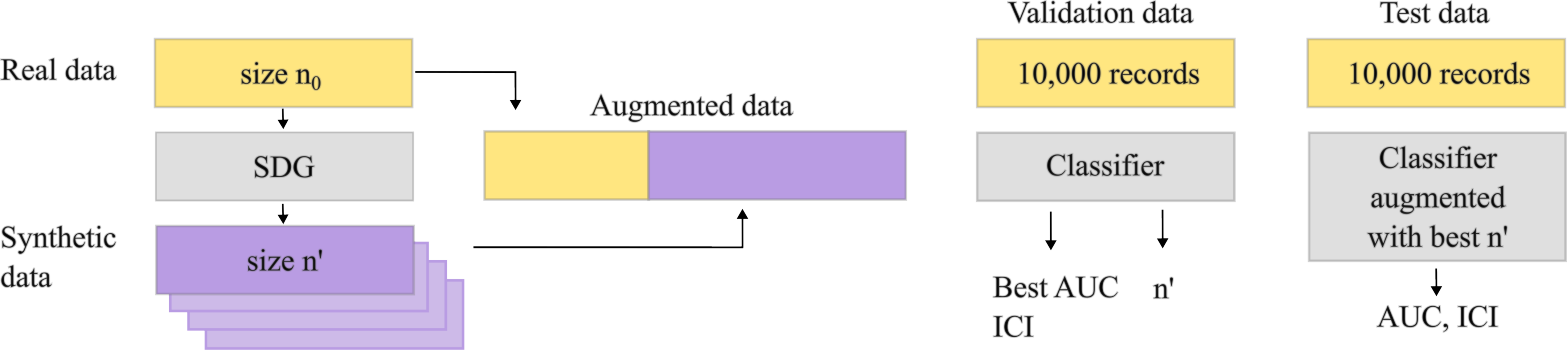
Augmentation scheme. AUC: area under the curve; ICI: integrated calibration index; SDG: synthetic data generation.

To determine the optimal n′, we selected an augmentation scheme that samples finely at the low end and coarsely at the high end of the range. Ten geometric series were created and provided n′ values varying from 5 to 10,000 records. The sizes of these synthetic datasets follow a geometric series defined by n′ = [*b*^(^*^i^* ^+ 4)^], where *b*~N(1.5, 0.005) and *i*=1,...,25. This results in multiple augmented datasets of size n = n_0_ + n′ for each base dataset. For each n′, 5 synthetic datasets were generated and used to augment the real data and train classifiers. To reduce the impact of generative model stochasticity, we computed the average performance across these 5 augmented datasets. The same hold-out validation real datasets of 10,000 records served to determine the n′, yielding the best AUC scores, and we used the same hold-out test datasets of 10,000 records for model performance evaluation and comparison (Figure 1).

### Datasets

Table 2 summarizes the 13 health datasets used in the study. These datasets cover heterogeneous domains, including public health, hospital discharge, infant and maternal health, adverse events, intensive care unit, population health surveys, and insurance claims. The table provides an overview of the datasets and the number of variables included in the binary classification models used to predict the outcome. A detailed description of each preprocessed dataset and the binary workload used for modeling can be found in Multimedia Appendix 1. The number of predictor variables in the workloads is consistent with what is seen in the clinical prediction literature [3].

## Results

We report the results for each research question. Full details of the statistical analysis are provided in Section F in Multimedia Appendix 1.

### Q1: Impact of Pretrained vs Non-Pretrained Augmentation

We compared pretrained and non-pretrained SDG methods across the 13 datasets using 1-tailed paired permutation tests (Table 3).

**Table 3.** Pairwise permutation tests to determine the impact of pretrained augmentation against non-pretrained augmentation. *P* values were adjusted for multiple comparisons using the Holm-Bonferroni procedure.

Null hypothesis	Alternative	AUC^a^-based score		ICI^b^-based score	
		Mean difference	Corrected *P* value	Mean difference	Corrected *P* value
TabPFN^c^ with pretrained augmentation and non-pretrained augmentation performs similarly (n_0_=50)	Pretrained augmentation performs better (n_0_=50)	0.0024	.22	−0.0063	.97
LGBM^d^ with pretrained augmentation and non-pretrained augmentation performs similarly (n_0_=50)	Pretrained augmentation performs better (n_0_=50)	0.0206^e^	.02^e^	−0.0279	.97
TabPFN with pretrained augmentation and non-pretrained augmentation performs similarly (n_0_=350)	Pretrained augmentation performs better (n_0_=350)	0.0003	.41	0.0018	.57
LGBM with pretrained augmentation and non-pretrained augmentation performs similarly (n_0_=350)	Pretrained augmentation performs better (n_0_=350)	0.0106^e^	.02^e^	−0.0057	.99

a AUC: area under the curve.

b ICI: integrated calibration index.

c TabPFN: Tabular Prior-Data Fitted Network.

d LGBM: light gradient boosting machine.

e Values significant at an *α* level of .05.

For TabPFN, pretrained augmentation did not lead to meaningful changes in discrimination at either dataset size (n_0_=50: ΔAUC=0.0024, *P*=.22; n_0_=350: ΔAUC=0.0003, *P*=.41).

In contrast, LGBM showed consistent and statistically significant improvements in AUC with pretrained augmentation (n_0_=50: ΔAUC=0.0206, median AUC=0.67, (IQR:0.66-0.69), *P*=.02; n_0_=350: ΔAUC=0.0106, median AUC=0.73, (IQR:0.71-0.75), *P*=.02), corresponding to gains of approximately 1 to 2 percentage points. These improvements were consistent in direction across 12 of the 13 datasets.

For calibration, pretrained augmentation was associated with small reductions in ICI for both models at n_0_=50, but these effects were modest and not statistically significant after correction for multiple testing. At n_0_=350, calibration differences between pretrained and non–pretrained augmentation were negligible.

### Q2: Impact of Augmentation vs No Augmentation

We evaluated the effect of data augmentation compared to no augmentation across 13 datasets using paired permutation tests with Holm-Bonferroni correction (Table 4). Based on Q1, pretrained SDG methods were used for LGBM, while all augmentation strategies were retained for TabPFN.

**Table 4.** Pairwise permutation tests to determine the impact of augmentation against no augmentation. *P* values were adjusted for multiple comparisons using the Holm-Bonferroni procedure.

Null hypothesis	Alternative	AUC^a^-based score		ICI^b^-based score	
		Mean difference	Corrected *P* value	Mean difference	Corrected *P* value
TabPFN^c^ with and without augmentation performs the same (n_0_=50)	Augmented TabPFN performs better (n_0_=50)	0.0094	.10	0.0134^d^	.04^d^
LLM^e^-augmented LGBM^f^ performs the same as LGBM without augmentation (n_0_=50)	LLM-augmented LGBM performs better (n_0_=50)	0.0764^d^	<.001^d^	0.0191^d^	.003^d^
TabPFN with and without augmentation performs the same (n_0_=350)	Augmented TabPFN performs better (n_0_=350)	0.0011	.06	0.0041	.09
LLM-augmented LGBM performs the same as LGBM without augmentation (n_0_=350)	LLM-augmented LGBM performs better (n_0_=350)	0.0078^d^	.02^d^	0.0119	.09

a AUC: area under the curve.

b ICI: integrated calibration index.

c TabPFN: Tabular Prior-Data Fitted Network.

d Values significant at an *α* level of .05.

e LLM: large language model.

f LGBM: light gradient boosting machine.

For TabPFN, augmentation did not significantly affect discrimination at either dataset size (n_0_=50: ΔAUC=0.0094, *P*=.10; n_0_=350: ΔAUC=0.0011, *P*=.06), indicating minimal impact on AUC.

For LGBM, LLM-based augmentation resulted in statistically significant improvements in discrimination at both n_0_=50 (ΔAUC=0.0764, *P*<.001) and n_0_=350 (ΔAUC=0.0078, *P*=.02), with larger gains observed in the smaller datasets.

For calibration, augmentation significantly reduced ICI at n_0_=50 for both TabPFN (ΔICI=0.0134, *P*=.04) and LGBM (ΔICI=0.0191, *P*=.003). At n_0_=350, calibration differences between augmented and nonaugmented models were small and not statistically significant.

### Q3: Comparison of Predictive Models

We compared model performance using the best configurations identified in Q1 and Q2 (Table 5).

**Table 5.** Pairwise permutation tests to determine the best classifier. *P* values were adjusted for multiple comparisons using the Holm-Bonferroni procedure.

Null hypothesis	Alternative	AUC^a^-based score		ICI^b^-based score	
		Mean difference	Corrected *P* value	Mean difference	Corrected *P* value
LLM^c^-augmented LGBM^d^ and LLM classifier perform similarly (n_0_=50)	LLM-augmented LGBM performs better (n_0_=50)	0.0634^e^	.04^e^	0.0152	.31
Augmented TabPFN^f^ and LLM classifier perform similarly (n_0_=50)	TabPFN performs better (n_0_=50)	0.0951^e^	.03^e^	0.0764^e^	.03^e^
Augmented TabPFN and LLM-augmented LGBM perform similarly (n_0_=50)	TabPFN performs better (n_0_=50)	0.0317^e^	.04^e^	0.0612^e^	<.001^e^
LLM-augmented LGBM and LLM classifier perform similarly (n_0_=350)	LLM-augmented LGBM performs better (n_0_=350)	0.0288^e^	.04^e^	0.0383	.06
Augmented TabPFN and LLM classifier perform similarly (n_0_=350)	TabPFN performs better (n_0_=350)	0.0441^e^	.01^e^	0.0484	.08
Augmented TabPFN and LLM-augmented LGBM perform similarly (n_0_=350)	TabPFN performs better (n_0_=350)	0.0153^e^	.004^e^	0.0101^e^	.02^e^

a AUC: area under the curve.

b ICI: integrated calibration index.

c LLM: large language model.

d LGBM: light gradient boosting machine.

e Values significant at an *α* level of .05.

f TabPFN: Tabular Prior-Data Fitted Network.

In terms of discrimination, both LLM-augmented LGBM and augmented TabPFN significantly outperformed LLM classifiers at both dataset sizes (all *P*<.05). Augmented TabPFN further achieved a significantly higher AUC than LLM-augmented LGBM (n_0_=50: ΔAUC=0.0317, *P*=.04; n_0_=350: ΔAUC=0.0153, *P*=.004), with a median AUC of 0.75 across datasets (IQR:0.74-0.77).

For calibration, augmented TabPFN showed significantly lower ICI than LGBM at both n_0_=50 (ΔICI=0.0612, *P*<.001) and n_0_=350 (ΔICI=0.0101, *P*=.02). Compared to LLM classifiers, TabPFN also demonstrated better calibration at n_0_=50 (ΔICI=0.0764, *P*=.03), while differences at n_0_=350 were smaller and not statistically significant (*P*=.08).

LLM classifiers are, therefore, reported in a fixed (no augmentation) configuration, reflecting their feasible operating regime under current computational constraints.

### Post Hoc Analysis: Augmentation Strategies

To further investigate whether the observed benefits of augmentation on TabPFN were driven by increased sample size rather than the generation of synthetic data, we compared SDG methods with sampling with replacement.

We evaluated 2 comparisons: (1) LLM-based synthesis versus the best-performing augmentation method (SDG or LLM) and (2) sampling with replacement versus the best-performing augmentation method. The results are reported in Table 6.

**Table 6.** Post hoc 2-sided pairwise permutation test to compare augmenting Tabular Prior-Data Fitted Network (TabPFN) by sampling with replacement or by synthetic data generation (SDG) methods.

Null hypothesis and n_0_	AUC^a^-based score		ICI^b^-based score	
	Statistic	*P* value	Statistic	*P* value
LLM^c^-based synthesis vs SDG methods and LLM-based synthesis				
50	−0.0024	.45	−0.0063	.41
350	−0.0003	.82	0.0018	.57
Sampling with replacement vs SDG methods and LLM-based synthesis				
50	−0.0058	.31	0.0016	.74
350	−0.0014	.41	−0.0013	.82

a AUC: area under the curve.

b ICI: integrated calibration index.

c LLM: large language model.

No statistically significant differences were observed for either AUC or ICI at both dataset sizes (all *P*>.3). Effect sizes were also negligible (|ΔAUC|<0.006; |ΔICI|<0.007), reinforcing the absence of meaningful performance differences.

### Ranking of Methods

The pairwise permutation tests supported the following performance ordering in terms of ICI and AUC: TabPFN-augmented with resampling>LLM-augmented LGBM>LLM classifiers. As a post hoc validation, we additionally applied the Page trend test, a nonparametric procedure designed to evaluate ordered alternatives. This analysis provided supporting evidence for a monotonic trend in classifier performance for both ICI and AUC (Table 7), consistent with the ranking obtained from the permutation tests. Together, these results provide convergent evidence that classifier performance follows the hypothesized order.

**Table 7.** Post hoc Page trend test (*L* value) results to validate rank order of classifiers’ performance.^a^^,^^b^

n_0_	AUC^c^-based score		ICI^d^-based score	
	*L*	Corrected *P* value	*L*	Corrected *P* value
50	167	0.02	166	.03
350	172	<.001	165	.048

a Null hypothesis: Augmented Tabular Prior-data Fitted Network (TabPFN) with sampling, large language model (LLM)-augmented light gradient boosting machine (LGBM), and LLM perform similarly.

b Alternative: Augmented TabPFN with sampling>LLM-augmented LGBM>LLM.

c AUC: area under the curve.

d ICI: integrated calibration index.

### Computational Cost

Computational resource requirements constitute a critical point of differentiation between the approaches considered. LLMs typically require extensive optimization and fine-tuning, resulting in substantial computational overhead. In contrast, TabPFN operates in a zero-shot setting, thereby obviating the need for dataset-specific training and significantly reducing computational demands. This distinction is especially relevant in clinical research contexts, where access to high-performance computing resources may be limited. The comparison in Table 8 illustrates the marked relative differences in training and inference time across methods.

**Table 8.** Approximate training and inference time per dataset.

Model	Time needed to train one model on a dataset of 1000 samples and infer on 10,000 samples
TabPFN^a^	6 seconds on a GPU^b^
LLM^c^ (average between the 3 considered LLMs)	12 hours on a GPU
LGBM^d^	20 minutes on CPUs^e^

a TabPFN: Tabular Prior-Data Fitted Network.

b GPU: graphics processing unit.

c LLM: large language model.

d LGBM: light gradient boosting machine.

e CPU: central processing unit.

## Discussion

### Summary

Health datasets available for research are often small, limiting the development of robust and generalizable clinical prediction models. Data augmentation is commonly used to mitigate this issue, including SDG methods such as CTGAN, Bayesian network, TVAE, and sequential trees [8,61,62]. However, these approaches can overfit when trained on limited data. An alternative is to leverage pretrained models, such as LLMs, to generate synthetic data without relying solely on the small dataset at hand.

In this study, we evaluated pretrained SDG using LLMs alongside traditional SDG approaches across 13 health datasets of sizes 50 and 350. This choice is consistent with current clinical prediction practices, where median training dataset sizes can be as low as 20 with a median dataset size of around 600 records [3,9]. We assessed their impact on 2 classifiers (LGBM and TabPFN), focusing on both discrimination and calibration. We also compared these approaches to using LLMs directly as classifiers.

### Key Findings and Practical Interpretation

#### Augmentation Benefits Depend on the Model

Augmentation improved LGBM performance, particularly for very small datasets, with LLM-based augmentation yielding the largest gains. In contrast, TabPFN showed little to no improvement in discrimination from augmentation (Table 4). Additionally, pretrained augmentation provided measurable gains for LGBM but not for TabPFN (Table 3), suggesting that the pretraining of TabPFN already confers robustness in low-data settings.

For practitioners working with limited data, TabPFN can be used effectively with minimal augmentation effort, while LGBM may benefit from targeted augmentation when small performance gains are meaningful. In scenarios where large external test sets are unavailable, selecting the augmentation size through 10-fold cross-validation provides stable performance estimates (Figure C1 in Multimedia Appendix 1), supporting its use as a practical model selection strategy in small-data settings.

#### Simple Methods Can Be as Effective as Complex Ones

For TabPFN, neither SDG methods nor LLM-based augmentation outperformed simple sampling with replacement (Table 6). This indicates that the primary benefit of augmentation is increasing the effective sample size rather than generating novel synthetic patterns.

In resource-limited or regulated health care environments, simple resampling should be the default strategy. It is computationally trivial, fully transparent, and avoids the risks associated with black-box generative models.

#### LLMs Are Not Reliable as Standalone Classifiers in Low-Data Settings

Across all experiments, LLMs used directly as classifiers performed worse than both TabPFN and LGBM, particularly in calibration (Table 5), with TabPFN achieving the best overall performance. Ranking analysis further supported this ordering (Table 7). Poor calibration is especially problematic in clinical contexts where probability estimates inform decisions.

Deploying LLMs as predictive models in small clinical datasets is not advisable. Their use is better suited for augmentation or other supportive roles rather than direct prediction.

#### Calibration Improvements Are Limited but Important

Calibration improvements were observed primarily in the smallest datasets (Table 4), with no significant effects observed at larger sample sizes.

In small datasets, augmentation can still be valuable for improving the reliability of predicted probabilities, even when discrimination gains are modest.

#### Model Choice Matters More as Data Increases

At n_0_=350, augmentation effects were smaller, and differences between models became more important (Table 4).

When moderate data is available, selecting an appropriate model (eg, TabPFN vs LGBM) is more impactful than investing in complex augmentation strategies.

#### Trustworthy and Resource-Constrained AI Considerations

A central finding of this study is that complex SDG does not outperform simple, transparent alternatives in small-data clinical settings. The absence of measurable gains from SDG or LLM-based augmentation over resampling (Table 6) highlights that an increased sample size is the main driver of performance improvements. This has direct implications for trustworthy AI:

- Transparency: Sampling with replacement provides a clear and traceable data generation process, unlike LLMs or SDG methods.
- Safety: Avoiding black-box generation reduces the risk of introducing unrealistic or misleading synthetic patient data.
- Regulatory alignment: Simpler methods are easier to justify and validate in clinical and regulatory contexts.
- Efficiency: TabPFN requires seconds to run, compared to hours for LLMs, making it accessible in low-resource environments.

Similarly, TabPFN combines strong predictive performance (Table 5) with minimal computational requirements (Table 8), making it particularly suitable for environments with limited resources.

Although LLM classifiers were not evaluated under augmentation due to practical computational constraints, this reflects a realistic deployment limitation rather than an experimental omission. Unlike TabPFN and LGBM, LLM fine-tuning with varying synthetic sample sizes is computationally prohibitive at scale, requiring hundreds of graphics processing unit (GPU)–days (see Section D in Multimedia Appendix 1). As such, the comparison reflects practical usability under resource-constrained conditions rather than fully symmetric experimental tuning.

#### Fidelity and Role of Synthetic Data

The post hoc analysis suggests that improvements from augmentation are primarily driven by increased sample size rather than the generation of novel synthetic data (Table 6).

While we performed basic realism checks to ensure that synthetic records respect plausible clinical ranges and preserve key distributions, perfect fidelity to the original data is not required for predictive tasks. Prior work [63] has shown that out-of-population observations can be common in synthetic datasets without necessarily degrading predictive performance. Similarly, generative models may improve generalization by increasing data diversity [8].

Taken together, these findings indicate that the benefits of augmentation are more closely related to increased sample size and diversity than to the exact replication of the original data distribution.

### Limitations and Future Work

This study focused on binary classification tasks and may not generalize to other settings, such as regression or survival analysis. Although we evaluated multiple datasets, further validation across diverse clinical contexts is needed.

While we evaluated 13 heterogeneous health datasets, they may not represent the full diversity of clinical data environments. Datasets with high dimensionality, extreme class imbalance, or strong temporal structure may behave differently under augmentation strategies. External validation on additional real-world datasets is required to strengthen generalizability.

Our experimental design relied on relatively large hold-out test sets to ensure stable estimation of discrimination and calibration metrics. In many real-world health care settings, such large external test sets are unavailable. Although our cross-validation analysis suggests that model selection through 10-fold cross-validation provides comparable estimates, performance variability may be higher in practice, particularly in ultra-small datasets.

Some of the non-pretrained models that were used in our analysis, such as CTGAN and TVAE, used the default hyperparameters and did not undergo additional tuning. While this may have limited their performance, sensitivity analyses presented in Section B in Multimedia Appendix 1 revealed that attempting to tune these hyperparameters on very small datasets can lead to overfitting. Specifically, we observed that the multivariate Hellinger distance (which is a common synthetic data fidelity metric) between the data from the generative models and sampling with replacement was lower when tuning compared to using default parameters. The sampling with replacement dataset serves as a baseline, indicating a high rate of overfitting. The fact that the fidelity was higher to the overfitted data with hyperparameter tuning indicates that tuning results in datasets that are closer to resampled data, which is highly overfitted. This highlights a practical limitation: in low-data settings, more complex model tuning may be counterproductive, and default configurations—or simpler augmentation strategies—can yield more reliable and stable results.

To adapt pretrained LLMs for tabular data, we used a serialization method from previous studies [33,35] that places column names before values, thereby preserving contextual information. While this creates artificial sentences, fine-tuning is thought to mitigate this issue. We did not explore alternative serialization methods, which could be an area for future research. Future work should explore broader classes of models, alternative data modalities, and evaluation in real-world deployment settings.

## Supplementary material

10.2196/88678 Multimedia Appendix 1 Additional methodological details and results.

## Abbreviations

AUC: area under the curve
CTGAN: conditional tabular generative adversarial network
GPU: graphics processing unit
ICI: integrated calibration index
LGBM: light gradient boosting machine
LLM: large language model
LoRA: low-rank adaptation
SDG: synthetic data generation
TabPFN: Tabular Prior-Data Fitted Network
TVAE: tabular variational autoencoder
