# Supplementary material for: An Evaluation of Pretrained Generative Models for Augmenting Small Health Data: Comparative Modeling Study
Source: J Med Internet Res. 2026 Jun 15;28:e88678. doi: 10.2196/88678 (PMC13268432; doi:10.2196/88678)
Supplement: Multimedia Appendix 1 [file jmir-v28-e88678-s001.pdf]

Multimedia Appendix

An evaluation of pre-trained generative models for augmenting small health data

Table of Contents

A. Augmentation scheme details 2

B. Hyperparameters 2

C. Result for augmentation performance (AUC - ICI) 6

D. Computation time and resource requirements for augmenting LLM classifiers 11

E. Details on the datasets 12

F. Details on the statistical analysis procedure 37

G. Fidelity check 39

H. References 39

A. Augmentation scheme details

The study begins by dividing the population dataset into training and test sets. From the training set, we extract two base datasets of sizes n0 = 50 and 350 using outcome-stratified random sampling. For each base dataset, a generative model is trained to simulate synthetic records. The sizes of these synthetic datasets (n') follow a geometric series defined by n' = [b^(i+4)], where b ~ N(1.5, 0.005) and i = 1, ..., 25. Ten such series are created, resulting in multiple augmented datasets of size n = n0 + n' for each base dataset.

Each augmented dataset is used to train an ML model, with all models evaluated on the same initial test set. This approach enables a systematic exploration of dataset size and augmentation effects on classification model performance across two base sizes and various augmentation levels.

B. Hyperparameters

This section summarizes the hyperparameter configurations considered across models. For LGBM, we report the default values, search ranges, and transformations used during tuning, while TabPFN was evaluated using its default settings. For LLM-based approaches, we additionally provide architectural details and fine-tuning configurations.

Hyperparameter	Default	Lower bound	Upper bound	Transform
booster	1 (gbdt)	1 (gbdt)	2 (goss)	2^learning_rate
Max_depth	6	1	15	
Learning_rate	log2 (0.3)	-10	0	
Early_stopping_rounds	7	7	30	
Min_data_in_leaf	10	1	60	
Num_leaves	15	4	60	

Table B.1: The default values and ranges of hyperparameters in LGBM models

Model name	HuggingFace tag	Size (GB)	Size in parameters	Pretrained data
DistilGPT2	Distilgpt2	0.35	0.08B	General data
Llama-3.2 1B	meta-llama/Llama-3.2-1B	2.5	1B	General data
Llama-3.2 8B	TsinghuaC3I/Llama-3-8B-UltraMedical	16	8B	Ultramedical dataset

Table B.1: List of open-source models selected for this study.

Finetuning epochs	Batchsize	Finetuning method	Optimizer	Learning rate	Dropout rate
150	128	LoRA (rank=16, alpha=32)	AdamW	5e-5, linear schedule	0.05

Table B.3: Finetuning parameters for large language models used in the study.

To enable the application of LLMs to structured tabular data, a serialization procedure was employed to convert each record into a textual representation. The following subsections describe the preprocessing, training, and generation procedures.

Data serialization and fine-tuning

Each tabular training record was serialized into a single text sequence following the pattern: "*column name 1* is *value 1*, *column name 2* is *value 2*, …". Missing values were explicitly encoded using a reserved "missing" token. LLMs were fine-tuned using a standard causal language modeling objective on the serialized training data only.

Generation procedure

Synthetic record generation did not use a system prompt or task instruction. Instead, for each synthetic sample, a single feature–value pair was randomly selected from the empirical distribution of the training data and provided as the initial context. The fine-tuned model then autoregressively generated the remaining feature values following the learned feature order until a complete record was produced.

Sampling parameters

- Temperature: 0.1
- Top-p: 0.9
- Maximum sequence length: 150 tokens, sufficient to cover one full record
- Decoding strategy: nucleus sampling

Post-processing

Generated sequences were parsed according to the predefined schema. Records with missing fields, invalid types, or constraint violations were discarded. Continuous variables were clipped to the observed training-data range when necessary.

Stochasticity control

For each configuration, five independent synthetic datasets were generated to account for sampling variability.

Sensitivity analysis on CTGAN and TVAE with default parameters vs hypertuned parameters

Finally, we present a sensitivity analysis comparing default and hyperparameter-tuned configurations for CTGAN and TVAE. Model selection was guided by a combined utility–vulnerability objective, designed to balance predictive performance with privacy risk. Specifically, this objective integrates multivariate Hellinger distance as a measure of distributional fidelity with established attribute and membership disclosure risk metrics, as described in the consensus privacy metrics framework for synthetic data [1]. This analysis evaluates the extent to which hyperparameter optimization impacts the fidelity of the generated distributions, as measured by multivariate Hellinger distance.

	TVAE		CTGAN	
	Multivariate Hellinger between sampling with replacement and hypertuned parameters	Multivariate Hellinger between sampling with replacement and default parameters	Multivariate Hellinger between sampling with replacement and hypertuned parameters	Multivariate Hellinger between sampling with replacement and default parameters
Born	0.279	0.647	0.289	0.616
Bsa	0.190	0.874	0.200	0.893
California	0.522	0.798	0.515	0.828
CCHS	0.226	0.628	0.217	0.649
Covid	0.292	0.256	0.238	0.274
FAERS	0.388	0.483	0.399	0.486
Florida	0.367	0.601	0.354	0.611
Mimic	0.177	0.304	0.150	0.362
Newyork	0.126	0.726	0.144	0.741
Nexoid	0.292	0.770	0.256	0.813
Texas	0.177	0.582	0.157	0.631
Washington	0.286	0.606	0.261	0.622
Washington 2008	0.196	0.777	0.221	0.845

Table B.4: Analysis comparing default and hyperparameter-tuned configurations of TVAE and CTGAN across datasets, measured using multivariate Hellinger distance to the empirical distribution (n0=50).

	TVAE		CTGAN	
	Multivariate Hellinger between sampling with replacement and hypertuned parameters	Multivariate Hellinger between sampling with replacement and default parameters	Multivariate Hellinger between sampling with replacement and hypertuned parameters	Multivariate Hellinger between sampling with replacement and default parameters
Born	0.267	0.466	0.273	0.628
Bsa	0.161	0.960	0.140	0.920
California	0.499	0.797	0.474	0.847
CCHS	0.144	0.632	0.130	0.696
Covid	0.278	0.277	0.399	0.263
FAERS	0.374	0.436	0.412	0.625
Florida	0.341	0.587	0.317	0.741
Mimic	0.197	0.309	0.156	0.481
Newyork	0.072	0.857	0.082	0.803
Nexoid	0.231	0.776	0.193	0.770
Texas	0.100	0.616	0.093	0.714
Washington	0.202	0.603	0.296	0.691
Washington 2008	0.137	0.772	0.126	0.760

Table B.5: Analysis comparing default and hyperparameter-tuned configurations of TVAE and CTGAN across datasets, measured using multivariate Hellinger distance to the empirical distribution (n0=350)

C. Result for augmentation performance (AUC - ICI)

Dataset	n0	LGBM trained on n0	TabPFN trained on n0	LLM finetuned on n0	LGBM trained on data augmented by ML models			LGBM trained on data augmented by LLM models			TabPFN fitted on data augmented by ML models			TabPFN fitted on data augmented by LLM models		
		AUC	AUC	AUC	Best method	n’	AUC	Best method	n’	AUC	Best method	n’	AUC	Best method	n’	AUC
BORN	50	0.517	0.890	0.899	BN	17	0.884	8B	29	0.899	SEQ	45	0.898	distilGPT2	6	0.892
	350	0.801	0.898	0.874	BN	492	0.910	1B	301	0.900	BN	1492	0.904	distilGPT2	42	0.899
BSA	50	0.495	0.667	0.650	TVAE	701	0.601	1B	780	0.602	CTGAN	9	0.674	8B	9	0.678
	350	0.653	0.719	0.719	CTGAN	5529	0.671	8B	7273	0.707	CTGAN	11	0.720	distilGPT2	29	0.727
California	50	0.656	0.760	0.572	SEQ	6	0.751	distilGPT2	17	0.734	SEQ	16	0.769	8B	10	0.763
	350	0.782	0.793	0.657	SEQ	1234	0.801	1B	45	0.798	SEQ	262	0.806	distilGPT2	276	0.813
CCHS	50	0.487	0.581	0.532	CTGAN	49	0.621	1B	4555	0.620	CTGAN	49	0.625	8B	6	0.611
	350	0.659	0.657	0.648	BN	7	0.659	8B	68	0.660	SEQ	170	0.667	8B	29	0.665
Covid	50	0.929	0.943	0.788	CTGAN	1054	0.934	1B	7	0.930	BN	11	0.944	8B	8	0.944
	350	0.943	0.945	0.916	CTGAN	211	0.942	distilGP2	19	0.941	CTGAN	23	0.945	1B	5	0.945
FAERS	50	0.524	0.503	0.529	BN	211	0.593	8B	2695	0.608	TVAE	8814	0.591	8B	5938	0.593
	350	0.582	0.598	0.562	BN	6	0.632	1B	190	0.618	SEQ	114	0.609	1B	73	0.605
Florida	50	0.633	0.762	0.562	CTGAN	11	0.771	distilGPT2	23	0.804	CTGAN	17	0.758	distilGPT2	32	0.769
	350	0.868	0.910	0.693	BN	1054	0.896	8B	374	0.900	SEQ	45	0.912	8B	73	0.912
MIMIC	50	0.579	0.503	0.632	TVAE	45	0.594	1B	780	0.632	BN	1797	0.531	1B	45	0.525
	350	0.569	0.666	0.684	SEQ	172	0.644	1B	19	0.640	TVAE	16	0.667	1B	414	0.663
New York	50	0.732	0.811	0.509	BN	13	0.782	8B	35	0.786	BN	23	0.814	8B	9	0.811
	350	0.822	0.848	0.642	SEQ	525	0.835	8B	172	0.839	SEQ	6	0.852	1B	45	0.851
Nexoid	50	0.520	0.578	0.553	BN	190	0.579	8B	1492	0.562	SEQ	72	0.594	distilGPT2	180	0.607
	350	0.637	0.666	0.606	CTGAN	5	0.645	8B	42	0.641	SEQ	262	0.682	8B	146	0.686
Texas	50	0.682	0.720	0.682	SEQ	2515	0.721	distilGPT2	3895	0.724	CTGAN	45	0.732	1B	80	0.729
	350	0.738	0.759	0.753	SEQ	507	0.732	distilGPT2	185	0.732	TVAE	1071	0.759	8B	10	0.759
Wash	50	0.624	0.656	0.616	CTGAN	1071	0.654	8B	439	0.662	BN	6	0.659	8B	23	0.665
	350	0.642	0.664	0.660	TVAE	584	0.665	distilGPT2	3895	0.685	SEQ	35	0.667	distilGPT2	342	0.689
Wash2008	50	0.638	0.629	0.545	TVAE	15	0.685	1B	16	0.691	TVAE	8	0.698	1B	11	0.672
	350	0.753	0.807	0.591	SEQ	757	0.778	8B	424	0.793	SEQ	8	0.809	8B	7	0.810

Table C.1: Comparison for the 13 datasets of the baseline LGBM trained on n0 real data, LGBM trained on augmented data generated by ML models described in Liu et al [], LGBM trained on augmented data generated by LLM and TabPFN fitted on n0 real data. In bold, the highest AUC score is highlighted and methods with a higher AUC score than the baseline (LGBM trained on n0 real data) is indicated by an underline. n’ indicates the number of synthetic samples yielding the corresponding AUC score.

Dataset	n0	LGBM trained on n0	TabPFN trained on n0	LLM finetuned on n0	LGBM trained on data augmented by non pretrained models			LGBM trained on data augmented by pretrained models			TabPFN fitted on data augmented by non pretrained SDG			TabPFN fitted on data augmented by pretrained models		
		ICI	ICI	ICI	Best method	n’	ICI	Best method	n’	ICI	Best method	n’	ICI	Best method	n’	ICI
BORN	50	0.460	0.038	0.040	SEQ	3603	0.050	8B	35	0.049	TVAE	11	0.025	1B	8814	0.020
	350	0.033	0.018	0.026	SEQ	1473	0.019	1B	73	0.018	SEQ	2993	0.012	8B	5	0.018
BSA	50	0.486	0.055	0.328	CTGAN	1071	0.030	8B	219	0.132	BN	185	0.037	8B	7	0.034
	350	0.045	0.047	0.273	CTGAN	3590	0.027	distilGPT2	62	0.030	SEQ	130	0.026	distilGPT2	65	0.020
California	50	0.162	0.055	0.243	SEQ	62	0.052	8B	108	0.066	BN	11	0.033	8B	13	0.042
	350	0.030	0.039	0.347	CTGAN	557	0.026	distilGPT2	77	0.042	SEQ	905	0.032	1B	19	0.035
CCHS	50	0.454	0.052	0.422	SEQ	1234	0.072	8B	7	0.076	TVAE	7	0.031	8B	5	0.042
	350	0.048	0.024	0.265	BN	73	0.025	distilGPT2	8	0.033	BN	10	0.019	8B	8	0.021
Covid	50	0.143	0.039	0.189	CTGAN	7860	0.042	1B	1054	0.056	BN	17	0.032	distilGPT2	6	0.041
	350	0.043	0.024	0.063	TVAE	8	0.025	8B	159	0.024	BN	7	0.021	8B	85	0.018
FAERS	50	0.191	0.026	0	CTGAN	849	0.026	1B	90	0.058	SEQ	2293	0.012	8B	5938	0.016
	350	0.132	0.020	0	TVAE	401	0.020	distilGPT2	439	0.022	SEQ	35	0.015	distilGPT2	90	0.017
Florida	50	0.431	0.112	0.173	SEQ	35	0.101	8B	116	0.104	CTGAN	9	0.056	8B	24	0.056
	350	0.051	0.036	0.349	CTGAN	4000	0.023	1B	64	0.030	CTGAN	46	0.032	1B	90	0.032
MIMIC	50	0.232	0.044	0	TVAE	211	0.053	1B	52	0.032	TVAE	24	0.036	8B	90	0.024
	350	0.092	0.065	0	CTGAN	90	0.029	distilGPT2	17	0.031	BN	180	0.040	1B	6334	0.015
New York	50	0.399	0.091	0.273	TVAE	1473	0.029	1B	1013	0.046	TVAE	1118	0.028	8B	42	0.033
	350	0.022	0.047	0.294	TVAE	20	0.016	1B	35	0.012	SEQ	1119	0.024	8B	243	0.023
Nexoid	50	0.478	0.135	0.566	CTGAN	159	0.082	8B	35	0.127	CTGAN	52	0.056	distilGPT2	5	0.082
	350	0.073	0.030	0.253	SEQ	17	0.040	1B	7	0.034	SEQ	6	0.030	8B	90	0.030
Texas	50	0.121	0.152	0.195	CTGAN	16	0.084	8B	67	0.067	CTGAN	29	0.090	1B	49	0.125
	350	0.032	0.38	0.188	CTGAN	2012	0.023	8B	45	0.029	CTGAN	5938	0.031	8B	23	0.035
Wash	50	0.049	0.045	0.343	SEQ	275	0.053	distilGPT2	72	0.088	SEQ	13	0.032	8B	8	0.040
	350	0.051	0.053	0.322	BN	371	0.034	1B	13	0.040	TVAE	254	0.028	distilGPT2	19	0.050
Wash2008	50	0.395	0.056	0.249	SEQ	1492	0.046	1B	62	0.073	SEQ	64	0.039	1B	11	0.053
	350	0.051	0.026	0.090	TVAE	3590	0.029	1B	8	0.036	TVAE	17	0.022	1B	33	0.021

Table C.2: Comparison for the 13 datasets of the baseline LGBM trained on n0 real data, LGBM trained on augmented data generated by ML models described in Liu et al [], LGBM trained on augmented data generated by LLM and TabPFN fitted on n0 real data. In bold, the lowest ICI score is highlighted and methods with a lower ICI score than the baseline (LGBM trained on n0 real data) is indicated by an underline. n’ indicates the number of synthetic samples yielding the corresponding ICI score.

Dataset	n0	TabPFN trained on n0	TabPFN fitted on data augmented by ML models			TabPFN fitted on data augmented by LLM models			TabPFN sampling with replacement	
		AUC	Best method	n’	AUC	Best method	n’	AUC	n’	AUC
BORN	50	0.890	SEQ	45	0.898	distilGPT2	6	0.892	62	**0.906**
	350	0.898	BN	1492	0.904	distilGPT2	42	0.899	85	**0.908**
BSA	50	0.667	CTGAN	9	0.674	8B	9	**0.678**	6	0.676
	350	0.719	CTGAN	11	0.720	distilGPT2	29	**0.727**	12	0.718
California	50	0.760	SEQ	16	**0.769**	8B	10	0.763	5	0.751
	350	0.793	SEQ	262	0.806	distilGPT2	276	**0.813**	23	0.800
CCHS	50	0.581	CTGAN	49	**0.625**	8B	6	0.611	9	0.572
	350	0.657	SEQ	170	0.667	8B	29	0.665	35	**0.671**
Covid	50	0.943	BN	11	**0.944**	8B	8	**0.944**	6	**0.944**
	350	**0.945**	CTGAN	23	**0.945**	1B	5	**0.945**	11	0.944
FAERS	50	0.503	TVAE	8814	0.591	8B	5938	**0.593**	131	0.581
	350	0.598	SEQ	114	**0.609**	1B	73	0.605	42	0.601
Florida	50	0.762	CTGAN	17	0.758	distilGPT2	32	**0.769**	6	0.751
	350	0.910	SEQ	45	**0.912**	8B	73	**0.912**	13	0.911
MIMIC	50	0.503	BN	1797	0.531	1B	45	0.525	170	**0.562**
	350	0.666	TVAE	16	**0.667**	1B	414	0.663	12	0.661
New York	50	0.811	BN	23	**0.814**	8B	9	0.811	5	**0.814**
	350	0.848	SEQ	6	**0.852**	1B	45	0.851	45	0.851
Nexoid	50	0.578	SEQ	72	0.594	distilGPT2	180	**0.607**	62	0.599
	350	0.666	SEQ	262	0.682	8B	146	**0.686**	35	0.685
Texas	50	0.720	CTGAN	45	**0.732**	1B	80	0.729	8	0.719
	350	**0.759**	TVAE	1071	**0.759**	8B	10	**0.759**	7	0.758
Wash	50	0.656	BN	6	0.659	8B	23	**0.665**	8	0.654
	350	0.664	SEQ	35	0.667	distilGPT2	342	**0.689**	45	0.671
Wash2008	50	0.629	TVAE	8	0.698	1B	11	0.672	16	**0.712**
	350	0.807	SEQ	8	0.809	8B	7	**0.810**	42	0.798

Table C.3: TabPFN augmentation comparison best AUC. Bold is highest value; underline is higher than baseline (no augmentation)

Dataset	n0	TabPFN trained on n0	TabPFN fitted on data augmented by non pretrained SDG			TabPFN fitted on data augmented by pretrained models			TabPFN sampling with replacement	
		ICI	Best method	n’	ICI	Best method	n’	ICI	n’	ICI
BORN	50	0.038	TVAE	11	0.025	1B	8814	**0.020**	7860	0.025
	350	0.018	SEQ	2993	**0.012**	8B	5	0.018	32	0.019
BSA	50	0.055	BN	185	0.037	8B	7	**0.034**	6	0.038
	350	0.047	SEQ	130	0.026	distilGPT2	65	**0.020**	24	**0.020**
California	50	0.055	BN	11	0.033	8B	13	0.042	11	**0.032**
	350	0.039	SEQ	905	**0.032**	1B	19	0.035	68	**0.032**
CCHS	50	0.052	TVAE	7	**0.031**	8B	5	0.042	5	0.054
	350	0.024	BN	10	0.019	8B	8	0.021	32	**0.018**
Covid	50	0.039	BN	17	**0.032**	distilGPT2	6	0.041	16	0.037
	350	0.024	BN	7	0.021	8B	85	**0.018**	24	0.025
FAERS	50	0.026	SEQ	2293	**0.012**	8B	5938	0.016	8	0.019
	350	0.020	SEQ	35	**0.015**	distilGPT2	90	0.017	6	0.021
Florida	50	0.112	CTGAN	9	0.056	8B	24	0.056	1786	**0.047**
	350	0.036	CTGAN	46	0.032	1B	90	0.032	374	**0.030**
MIMIC	50	0.044	TVAE	24	0.036	8B	90	**0.024**	6	0.045
	350	0.065	BN	180	0.040	1B	6334	**0.015**	108	0.032
New York	50	0.091	TVAE	1118	**0.028**	8B	42	0.033	35	0.050
	350	0.047	SEQ	1119	0.024	8B	243	**0.023**	6	0.028
Nexoid	50	0.135	CTGAN	52	**0.056**	distilGPT2	5	0.082	864	0.103
	350	0.030	SEQ	6	**0.030**	8B	90	**0.030**	17	0.034
Texas	50	0.152	CTGAN	29	**0.090**	1B	49	0.125	557	0.106
	350	0.038	CTGAN	5938	**0.031**	8B	23	0.035	11	0.034
Wash	50	0.045	SEQ	13	0.032	8B	8	0.040	14	**0.026**
	350	0.053	TVAE	254	**0.028**	distilGPT2	19	0.050	64	0.050
Wash2008	50	0.056	SEQ	64	**0.039**	1B	11	0.053	6	0.067
	350	0.026	TVAE	17	0.022	1B	33	**0.021**	347	0.053

Table C.4: TabPFN augmentation comparison best ICI. Bold is lowest value, underline is lower than baseline (no augmentation)

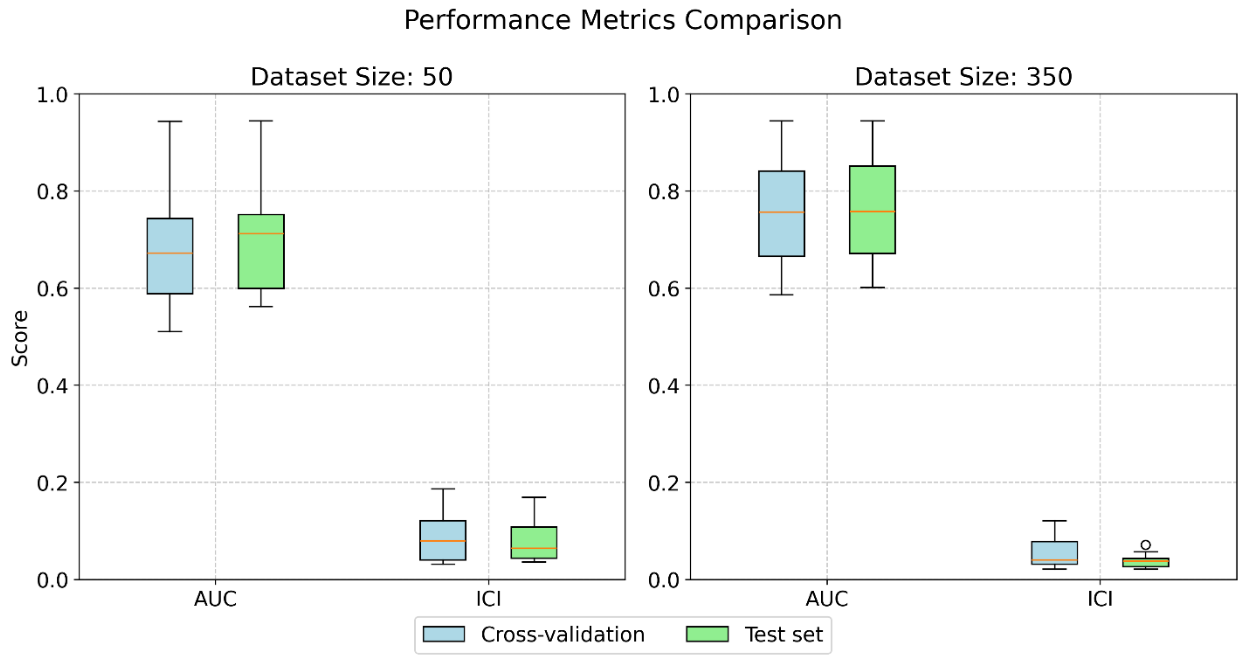

Figure C.1: TabPFN augmented with n’ sampled with replacement records where n’ is determined on the test set or on the 10-fold cross validation.

D. Computation time and resource requirements for augmenting LLM classifiers

The computational resources required for augmenting LLM classifiers were found to be prohibitively extensive, necessitating their exclusion from the main study. This section outlines the scale of the computational challenge to justify this decision.

For each of the three LLMs considered in this study, the process of simulating various degrees of augmentation using four non-pretrained Synthetic Data Generation (SDG) models across different datasets would result in approximately 100,000 individual training instances. This estimate is derived from the following parameters:

- Number of datasets: 13
- Number of base dataset sizes: 2
- Number of non-pretrained SDG models: 4
- Number of synthetic data sequences: 10
- Number of points in each sequence: 19
- Number of iterations: 5

Given that a single training instance on one GPU requires an average of 11 hours, the total computational time for this endeavor becomes substantial. Even with access to a maximum of 15 GPUs, the complete process would necessitate approximately 572 days of continuous computation.

This time frame is clearly impractical for the scope of this study and would be unrealistic for most practitioners. The extensive computational demands underscore the challenges of applying such augmentation techniques at scale and highlight the need for more efficient methods in future research.

The exclusion of these results from the main paper thus reflects both practical constraints and the recognition that such resource-intensive approaches may not be feasible in many real-world applications.

E. Details on the datasets

The following are the details for the preprocessed datasets that were used prior to model training in this study.

Better Outcomes Registry & Network

Data are collected from the BORN Ontario birth registry that covers about 1 million records regarding Ontario's maternal demographic characteristics, obstetrical history, health behaviors, prenatal screening and newborn care information. We combine the pregnancy and infant datasets and examine the association between low birthweight and its related risk factors. The relevant factors include gestational age, maternal age, maternal body mass index, total number of pregnancies a mother has experienced, number of previous preterm pregnancies, number of previous abortions, maternal smoking status, alcohol exposure, prenatal screening, mental health concerns for addiction, anxiety, depression, maternal health conditions for diabetes and genetics and drug exposure to Cocaine, Hallucinogens and Opioids. We follow the definition of low birthweight[25] and classify the newborns whose birth weights are less than 2,500 grams as infants with low birthweight. A value of 1 is given for newborns with low birthweight and 0 otherwise. A summary of descriptive statistics for the variables is presented in Table E.1.

Variable	Description	Type	Mean (SD) or level count (% of total size) or number of categories	Missingness (% of the total size)
Birth weight	Whether a newborn has low birthweight (<2,500 g)	Categorical	1: 7.01%<br>0: 92.99%	0.00%
Gestational age	Gestational age of a newborn baby	Numeric	1 < 34 weeks: 2.32%<br>2 34-36 weeks: 6.00%<br>3 37-38 weeks: 27.15%<br>4 39-41 weeks: 64.11%<br>5 >=42 weeks: 0.43%	0.00%
Maternal age	Maternal age in years at time of stillbirth or live birth	Numeric	1 <= 19: 2.15%<br>2 20-34: 74.12%<br>3 35-39: 19.31%<br>4 >=40: 4.36%	0.06%
Maternal BMI	Maternal pre-pregnancy body mass index	Numeric	1 <18.5: 4.53%<br>2 18.5-24.9: 43.44%<br>3 25-29.9: 20.22%<br>4 >=30: 15.86%	15.94%
Parity	Total number of pregnancies a mother has experienced	Numeric	0: 42.82%<br>1: 34.57%<br>2: 13.98%<br>3: 4.64%<br>>=4: 2.89%	1.10%
Preterm birth	Number of previous preterm pregnancies	Numeric	0: 93.57%<br>1: 4.41%<br>2: 0.71%<br>3: 0.13%<br>>=4: 0.05%	1.13%
Abortions	Number of previous abortions	Numeric	0: 65.77%<br>1: 20.85%<br>2: 7.25%<br>3: 2.50%<br>>=4: 1.50%	2.13%

Smoking	Maternal smoking status at time of admission	Categorical	Yes: 7.64%<br>No: 88.14%	4.21%
Alcohol	Alcohol exposure in pregnancy	Categorical	Yes: 2.23%<br>No: 92.54%	5.23%
Prenatal screening	Whether a mother has prenatal screening during pregnancy	Categorical	Yes: 66.30%<br>No: 33.70%	0.00%
Addiction	Mental health concern regarding addiction	Categorical	Yes: 0.60%<br>No: 93.72%	5.68%
Anxiety	Mental health concern regarding anxiety	Categorical	Yes: 8.99%<br>No: 85.33%	5.68%
Depression	Mental health concern regarding depression	Categorical	Yes: 7.58%<br>No: 86.74%	5.68%
Diabetes	Maternal health condition regarding diabetes	Categorical	Yes: 1.00%<br>No: 93.27%	5.73%
Genetics	Maternal health condition regarding genetics	Categorical	Yes: 0.00%<br>No: 94.26%	5.73%
Cocaine drug	Drug exposure to Cocaine in pregnancy	Categorical	Yes: 0.25%<br>No: 94.65%	5.10%
Hallucinogens drug	Drug exposure to Hallucinogens in pregnancy	Categorical	Yes: 0.02%<br>No: 94.88%	5.10%
Opioids drug	Drug exposure to Opioids in pregnancy	Categorical	Yes: 0.42%<br>No: 94.48%	5.10%

Note: SD: standard deviation

Table E.1: Descriptive statistics for the BORN dataset.

Basic Stand Alone Inpatient Claims

This dataset contains the claim-level information with each recording being an inpatient claim chosen from a 5% random sample of Medicare beneficiaries during 2008. In this study, we choose the variables including age, gender, DRG, ICD-9 primary procedure code, Medicare payment and the length of stay and explore the relationship between the length of stay and its relevant demographic and claim-related factors. The outcome is defined as a binary variable taking a value of 1 if the length of stay on the file is greater than or equal to 2.5 days, and 0 otherwise. **Table E.2** provides an overview of the detailed statistics for these variables.

Variable	Description	Type	Mean (SD) or 1 count (% of total size) or number of categories	Missingness (% of the total size)
Outcome	Whether the length of stay on a claim is greater than 2.5 days	Categorical	1: 42.65%<br>0: 57.35%	0.00%
Age	The beneficiary's age	Numeric	1 Under 65: 19.73%<br>2 65- 69: 13.19%<br>3 70-74: 14.65%<br>4 75-79: 15.55%<br>5 80-84: 16.10%<br>6 85 & older: 20.78%	0.00%
Gender	The beneficiary's gender	Categorical	1 Male: 43.88%<br>2 Female: 56.12%	0.00%
DRG	Diagnostic related groups to which a hospital claim belongs for prospective payment purposes	Categorical	311	0.00%
ICD-9	Primary procedure (primarily surgical procedures) performed during the inpatient stay	Categorical	86	47.00%

Payment	Quintile value (or code) to which the actual Medicare payment amount on the claim belongs	Categorical	1: 19.97%<br>2: 20.12%<br>3: 20.00%<br>4: 19.82%<br>5: 20.09%	0.00%

Note: SD: standard deviation

Table E.2: Descriptive statistics for the BSA dataset.

California Hospital Discharges

This dataset contains over 4 million inpatient discharge records in 2008 from community hospitals in California from State Inpatient Databases that are used to track the trends in healthcare utilization, access, charges, quality and outcomes in United States. We are interested in exploring the relationship between length of stay and its demographic and health factors. Specifically, the covariates of interest include age, female, race, aweekend, DRG, DX1, primary payer, total charges, chronic conditional indicators, and procedure classes for ICD-10-PCS procedure codes, comorbidity measures for alcohol abuse, depression, hypertension and obesity. The outcome is generated by dividing the patients into two groups based on the median of their length of stay. A value of 1 is assigned if the patient's length of stay is greater than or equal to 3 days and 0 otherwise. Detailed statistics of the variables are displayed in **Table E.3**.

**Variable**	**Description**	**Type**	**Mean (SD) or level count (% of total size) or number of categories**	**Missingness (% of the total size)**
Outcome	Whether a patient's length of stay is greater than 3 days	Categorical	1: 54.10%<br>0: 45.90%	0.00%
AGE	Patient's age in years	Numeric	44.59 (28.58)	0.95%
FEMALE	Whether a patient's gender is female	Categorical	1 Female: 57.24%<br>0 Male: 39.85%	2.91%
RACE	Patient's race	Categorical	1 White: 46.72%<br>2 Black: 7.27%	8.31%

			3 Hispanic: 28.52%<br>4 Asian or Pacific Islander: 7.07%<br>5 Native American: 0.07%<br>6 Other: 2.04%	
AWEEKEND	Whether a patient's admission day is on a weekend	Categorical	1 Admitted Saturday - Sunday: 20.39%<br>0 Admitted Monday - Friday: 79.61%	0.00%
DRG	Diagnosis Related Group	Categorical	746	0.00%
DX1	ICD-9-CM Diagnosis	Categorical	8,548	0.00%
PAY1	Expected primary payer (Medicare, Medicaid, private insurances, etc.)	Categorical	1 Medicare: 31.13%<br>2 Medicaid: 25.57%<br>3 Private insurance: 34.79%<br>4 Self-pay: 3.41%<br>6 Other: 5.09%	0.02%
TOTCHG	Total charges	Numeric	45,065.28 (78,294.38)	12.00%
CHRON1	ICD-9-CM Chronic Condition Indicators	Categorical	1 Chronic condition: 34.22%<br>0 Non-chronic condition: 65.78%	0.00%
CHRONB1	Chronic Condition Indicators - body system	Categorical	19	0.00%
PCLASS1	Procedure Classes Refined for ICD-10-PCS procedure codes	Categorical	1 Minor diagnostic: 9.65%<br>2 Minor therapeutic: 28.12%<br>3 Major diagnostic: 0.46%<br>4 Major Therapeutic: 26.37%	35.40%

CM_ALCOH	AHRQ comorbidity measure for ICD-9-CM codes: alcohol abuse	Categorical	1 Comorbidity is present: 3.84%<br>0 Comorbidity is not present: 96.16%	0.00%
CM_DEPRE	AHRQ comorbidity measure for ICD-9-CM codes: depression	Categorical	1 Comorbidity is present: 5.80%<br>0 Comorbidity is not present: 94.20%	0.00%
CM_HTN_C	AHRQ comorbidity measure for ICD-9-CM codes: hypertension (combine uncomplicated and complicated)	Categorical	1 Comorbidity is present: 33.31%<br>0 Comorbidity is not present: 66.69%	0.00%
CM_OBESE	AHRQ comorbidity measure for ICD-9-CM codes: obesity	Categorical	1 Comorbidity is present: 7.23%<br>0 Comorbidity is not present: 92.77%	0.00%

Note: SD: standard deviation

Table E.3: Descriptive statistics for the hospital California dataset.

Canadian Community Health Survey

The CCHS data is a cross-sectional telephone survey administered by Statistics Canada that collects information on the health status, health care utilization and health determinants of Canadians. This dataset is a pooled version of survey data from 2001 to 2013, and the variables we are using are presented in Table E.4.

The model outcome is cardiovascular health and the covariates are age, sex, education, house income, household size, and immigration as predictors to predict the ideal state of cardiovascular health using variables from the dataset [2]. To assess cardiovascular health, we follow the definition of ideal cardiovascular health introduced by the American Heart Association to calculate the Cardiovascular Health in Ambulatory Care Research Team (CANHEART) health index score, which is determined by 7 health factors including smoking, obesity, hypertension, diabetes, physical activity, and fruit and vegetable consumption [3]. The

final CANHEART index score ranges from 0 (worst) to 6 (best). The outcome is assigned to be 1 if the score is above 3 [4], which is considered to be an intermediate or ideal state of cardiovascular health and 0 otherwise.

**Variable**	**Description**	**Type**	**Mean (SD) or level count (% of total size)**	**Missingness (% of the total size)**
CANHEART	Whether a patient is in ideal cardiovascular health; this is a sum of the prior six variables.	Categorical	1 Ideal: 63.95%<br>0 Non-ideal: 36.05%	0.00%
Age	Patient’s age in years	Numeric	47.24 (20.19)	0.00%
Sex	Patient’s gender	Categorical	1 Male: 45.84%<br>2 Female: 54.16%	0.00%
Education	Patient’s highest level of education	Categorical	1 < Secondary school graduate: 26.14%<br>2 Secondary school graduate: 16.93%<br>3 Some post-secondary education: 6.65%<br>4 Post-secondary certificate: 48.55%	1.72%
Marital status	Patient’s marital status	Categorical	1 Married: 43.41%<br>2 Common-law: 8.12%<br>3 Widow/separation /divorce: 19.55%<br>4 Single/never married: 28.74%	0.17%
House income	Total household income from all sources	Numeric	57,603.42 (32,061.49)	9.59%
Household size	Size of entire household	Numeric	2.39 (1.23)	15.66%

Immigration	Whether a patient is an immigrant	Categorical	1 Yes: 13.86%<br>2 No: 84.33%	1.81%

Note: SD: standard deviation

Table E.4: Descriptive statistics for the CCHS dataset.

Canadian COVID-19

The first dataset is the Canadian COVID-19 dataset from the Public Health Agency of Canada. It contains over 1 million health records of individuals who have tested positive for COVID-19. We are interested in fitting a model that predicts mortality caused by COVID-19. The binary outcome of interest is derived from the case status in the dataset, and a value of 1 is assigned if the patient has died due to COVID-19 while a value 0 is assigned if the patient has recovered. The selected predictors for modeling include the following variables: date, age group, gender, region, exposure, province. **Table E.5** presents an overview of the variables that are included in the binary model.

Variable	Description	Type	Mean (SD) or level count (% of total size) or number of categories	Missingness (% of the total size)
Case status	The status of a patient	Categorical	1: 1.48 %<br>0: 98.52%	0.00%
Date	The date when a case is reported	Numeric (computed as the number of days since 1st January 2020)	348.07 (96.16)	0.00%
Age group	Patient's age group in years	Numeric	1 <20: 17.92%<br>2 20-29: 20.26%<br>3 30-39: 17.08%<br>4 40-49: 14.82%<br>5 50-59: 13.53%<br>6 60-69: 8.26%<br>7 70-79: 3.99%<br>8 >80: 2.16%	1.99%

Gender	Patient's gender	Categorical	Female: 49.39%<br>Male: 50.05%	0.56%
Region	Health unit in Canada	Categorical	40	0.00%
Exposure	The type of being exposed to someone with COVID-19	Categorical	Close contact: 31.21%<br>Outbreak: 10.78%<br>Travel-related: 1.14%	56.87%
Province	Province in Canada where case is reported	Categorical	Ontario: 70.25%<br>Alberta: 29.75%	0.00%

Note: SD: standard deviation

Table E.5: Descriptive statistics for the COVID-19 dataset.

FDA Adverse Event Reporting System

The next dataset contains the reports submitted to the FDA Adverse Event Reporting System for patients with adverse events. The binary outcome of interest for this dataset is whether or a patient has died. Our primary goal with this dataset is to explore the relationship between patient mortality and various predictors, including event date, gender, age, weight, drug name and the indication for drug use. Detailed statistics for these variables can be found in **Table E.6**.

Variable	Description	Type	Mean (SD) or level count (% of total size) or number of categories	Missingness (% of the total size)
Outcome	Whether a patient has died	Categorical	1 Death: 9.94%<br>0 Non-death: 90.06%	0.00%
Event date	Date the adverse event occurred	Numeric (difference from 1/1/2020)	466.52 (827.94)	62.26%
Gender	Patient's gender	Categorical	Female: 51.82%<br>Male: 37.85%	10.33%

Age	Patient's age in years	Numeric	55.90 (20.80)	33.41%
Weight	Patient's weight in kg	Numeric	73.05 (25.70)	74.13%
Drug name	Name of medicinal product	Categorical	10,545	0.00%
Indication	Medical terminology describing the indication for use	Categorical	4,287	0.00%

Note: SD: standard deviation

Table E.6: Descriptive statistics for the FAERS dataset.

Florida Hospital Discharges

This dataset contains over 2.3 million inpatient discharge records in 2007 from community hospitals in Florida from State Inpatient Databases that are used to track the trends in healthcare utilization, access, charges, quality and outcomes in United States. We are interested in exploring the relationship between length of stay and its demographic and health factors. Specifically, the covariates of interest include age, female, race, admission type, aweekend, DRG, DX1, primary payer, total charges and zip code. The outcome is created by classifying the patients into two groups based on the median length of their stay. A value of 1 is assigned if the patient's length of stay is greater than or equal to 3 days and 0 otherwise. Detailed statistics of the variables are displayed in **Table E.7**.

Variable	Description	Type	Mean (SD) or level count (% of total size) or number of categories	Missingness (% of the total size)
Outcome	Whether a patient's length of stay is greater than 3 days	Categorical	1: 60.46%<br>0: 39.54%	0.00%
AGE	Patient's age in years	Numeric	51.23 (27.04)	0.00%
FEMALE	Whether a patient's gender is female	Categorical	1 Female: 56.02%<br>0 Male: 43.98%	0.00%
RACE	Patient's race	Categorical	1 White: 65.53%<br>2 Black: 16.92%<br>3 Hispanic: 13.28%<br>4 Asian or Pacific Islander: 0.76%<br>5 Native American: 0.27%<br>6 Other: 2.47%	0.76%
ATYPE	Admission type	Categorical	1 Emergency: 54.15%<br>2 Urgent: 16.43%<br>3 Elective: 20.73%<br>4 Newborn: 8.17%<br>5 Trauma Center: 0.52%	0.00%
AWEEKEND	Whether a patient's admission day is on a weekend	Categorical	1 Admitted Saturday - Sunday: 19.51%<br>0 Admitted Monday - Friday: 80.49%	0.00%
DRG	Diagnosis Related Group	Categorical	861	0.00%
DX1	ICD-9-CM Diagnosis	Categorical	7,380	0.00%

PAY1	Expected primary payer (Medicare, Medicaid, private insurances, etc.)	Categorical	1 Medicare: 42.71%<br>2 Medicaid: 17.50%<br>3 Private insurance: 27.52%<br>4 Self-pay: 6.23%<br>5 No charge: 2.28%<br>6 Other: 3.77%	0.00%
TOTCHG	Total charges	Numeric	33,604.48 (52,812.95)	0.01%
CHG1	All inclusive detailed charges	Numeric	1,818.47 (2,841.07)	0.00%
ZIP	Zip code	Categorical	14,728	0.64%

Note: SD: standard deviation

Table E.7: Descriptive statistics for the hospital Florida dataset.

Medical Information Mart for Intensive Care III

The dataset is extracted from the MIMIC-III relational database (version 1.4), which contains deidentified clinical data of the patients who were admitted to the Beth Israel Deaconess Medical Center in Boston, Massachusetts [5–7]. It contains various tables of patient data regarding demographics, admission information, lab tests, diagnosis codes, caregiver information, and discharge notes. We use this dataset to investigate the relationship between 30-day readmission and its related demographics, vital signs and lab test values. The demographics include the age of the patients when they were first admitted to the ICU, their ethnicity group and admission type. The vital signs consider the (systolic and diastolic) blood pressure, heart rate and respiration rate. Several lab measurements are also incorporated into the analysis. The selection criteria for readmitted patients are to include those who were readmitted within 30-day of initial hospital discharge from the ICU. The patients who were readmitted to the ICU are assigned a label of 1, while those who were not readmitted are assigned a label of 0. **Table E.8** summarizes the descriptive statistics of the selected variables.

Variable	Description	Type	Mean (SD) or level count (% of total size) or number of categories	Missingness (% of the total size)
Readmission	Whether a patient is re-admitted to ICU	Categorical	1 Yes: 21.01%<br>0 No: 78.99%	0.00%
Age	Patient's age in the time of first admission	Numeric	63.43 (16.16)	0.00%
Ethnicity	Patient's ethnicity group	Categorical	39	15.14%
Admission type	Patient's admission type	Categorical	Elective: 18.10%<br>Emergency: 78.52%<br>Urgent: 3.37%	0.00%
Heart rate	Vital sign for heart rate	Numeric	87.86 (15.89)	0.47%
NT-proBNP	Lab test for N-terminal prohormone of brain natriuretic peptide	Numeric	4.10 (1.17)	43.55%
Creatinine	Lab test for serum creatinine	Numeric	4.10 (1.17)	43.54%
Bun	Lab test for blood urea nitrogen	Numeric	4.10 (1.17)	43.53%
Potassium	Lab test for potassium	Numeric	4.10 (1.17)	43.52%
Cholesterol	Lab test for cholesterol	Numeric	4.10 (1.17)	43.54%

Note: SD: standard deviation

Table E.8: Descriptive statistics for the MIMIC-III dataset.

New York Hospital Discharges

This dataset consists of over 2.6 million inpatient discharge records in 2007 from community hospitals in New York from State Inpatient Databases that are used to track the trends in healthcare utilization, access, charges, quality and outcomes in the United States. We are interested in examining the relationship between length of stay and demographic and health factors. Specifically, the covariates of interest include age, female, race, admission type,

aweekend, DRG, DX1, primary payer, total charges, zip code, chronic conditional indicators and procedure classes for ICD-10-PCS procedure codes. The outcome is created by classifying the patients into two groups based on the median length of their stay. A value of 1 is assigned if the patient's length of stay is greater than or equal to 3 days and 0 otherwise. Detailed statistics of the variables are displayed in **Table E.9**.

Variable	Description	Type	Mean (SD) or level count (% of total size) or number of categories	Missingness (% of the total size)
Outcome	Whether a patient's length of stay is greater than 3 days	Categorical	1: 61.82%<br>0: 38.18%	0.00%
AGE	Patient's age in years	Numeric	48.87 (27.36)	0.00%
FEMALE	Whether a patient's gender is female	Categorical	1 Female: 56.68%<br>0 Male: 43.32%	0.00%
RACE	Patient's race	Categorical	1 White: 56.73%<br>2 Black: 17.43%<br>3 Hispanic: 13.56%<br>4 Asian or Pacific Islander: 3.38%<br>5 Native American: 1.01%<br>6 Other: 5.90%	1.98%
ATYPE	Admission type	Categorical	1 Emergency: 60.22%<br>2 Urgent: 9.86%<br>3 Elective: 20.86%<br>4 Newborn: 8.90%<br>5 Trauma Center: 0.00%	0.16%
AWEEKEND	Whether a patient's admission day is on a weekend	Categorical	1 Admitted Saturday - Sunday: 19.26%<br>0 Admitted Monday - Friday: 80.74%	0.00%
DRG	Diagnosis Related Group	Categorical	863	0.00%

DX1	ICD-9-CM Diagnosis	Categorical	7,956	0.00%
PAY1	Expected primary payer (Medicare, Medicaid, private insurances, etc.)	Categorical	1 Medicare: 36.08%<br>2 Medicaid: 23.69%<br>3 Private insurance: 32.38%<br>4 Self-pay: 5.44%<br>5 No charge: 0.17%<br>6 Other: 2.23%	0.00%
TOTCHG	Total charges	Numeric	24,628.84 (43,545.43)	0.01%
ZIP	Zip code	Categorical	10,814	0.00%
CHRON1	ICD-9-CM Chronic Condition Indicators	Categorical	1 Chronic condition: 40.86%<br>0 Non-chronic condition: 59.14%	0.00%
CHRONB1	Chronic Condition Indicators - body system	Categorical	19	0.00%
PCLASS1	Procedure Classes Refined for ICD-10-PCS procedure codes	Categorical	1 Minor diagnostic: 15.19%<br>2 Minor therapeutic: 31.30%<br>3 Major diagnostic: 0.54%<br>4 Major Therapeutic: 25.86%	27.12%

Note: SD: standard deviation

Table E.9: Descriptive statistics for the hospital New York dataset.

Nexoid COVID-19 Survival Calculator

The COVID-19 survival dataset that is used in the study is a web-based survey data collected by the research team by Nexoid, a company in the United Kingdom. They collect demographic, socioeconomic and health-related information of individuals to predict two crucial aspects related to COVID-19: the probability of being infected with COVID-19 as well as the probability of mortality associated with COVID-19. In our study, we focus on the probability of COVID-19

infection using important demographic, behavioral and health factors including age, sex, race, smoking, nursing home, COVID-19 symptoms, COVID-19 contact, health worker, and the presence of comorbidities such as asthma, kidney disease, liver disease, heart disease, lung disease, diabetes, and hypertension. The outcome of interest is determined by the risk scores of getting infected with COVID-19. The patients whose risk scores exceed the mean risk score are considered as having a high risk of contracting COVID-19, while those with scores below the mean are classified as having a low risk. **Table E.10** summarizes the basic statistics of the selected variables.

**Variable**	**Description**	**Type**	**Mean (SD) or level count (% of total size)**	**Missingness (% of the total size)**
Outcome	Whether a patient has a high risk of getting infected with COVID-19	Categorical	1 Yes: 39.20%<br>0 No: 60.80%	0.00%
Age	Age group in years	Numeric	1 0_10: 0.58%<br>2 10_20: 3.35%<br>3 20_30: 21.56%<br>4 30_40: 29.84%<br>5 40_50: 21.09%<br>6 50_60: 12.48%<br>7 60_70: 7.47%<br>8 70_80: 2.97%<br>9 80_90: 0.54%<br>10 90_100: 0.12%<br>11 100_110: 0.01%	0.00%
Sex	Patient's gender	Categorical	Female: 63.13%<br>Male: 36.53%	0.34%
Race	Patient's race	Categorical	White: 24.19%<br>Hispanic: 1.40%<br>Asian: 1.21%<br>Mixed: 0.96%<br>Black: 0.46%<br>Other: 0.32%	71.45%
Smoking	Type of smoking	Categorical	Heavy: 1.68%	0.19%

			Medium: 7.64%<br>Light: 4.44%<br>Quit0: 5.54%<br>Quit5: 6.58%<br>Quit10: 9.23%<br>Vape: 5.95%<br>Never smoked: 58.74%	
BMI	Body mass index	Numeric	29.37 (7.81)	0.00%
House count	House person count	Numeric	3.14 (1.57)	0.00%
Public transport count	Number of public transports used	Numeric	0.38 (1.70)	71.12%
Nursing home	Whether it is a nursing home	Categorical	1: 0.07%<br>0: 99.93%	0.00%
COVID-19 symptoms	Whether a patient shows symptoms of COVID-19	Categorical	1: 2.04%<br>0: 97.96%	0.00%
COVID-19 contact	Whether a patient has close contact with someone infected with COVID-19	Categorical	1: 4.33%<br>0: 95.67%	0.00%
Health worker	Whether a patient is a healthcare worker	Categorical	1: 1.79%<br>0: 98.21%	0.00%
Asthma	Whether a patient has asthma	Categorical	1: 15.26%<br>0: 84.74%	0.00%
Kidney disease	Whether a patient has kidney disease	Categorical	1: 0.36%<br>0: 99.64%	0.00%
Liver disease	Whether a patient has liver disease	Categorical	1: 0.21%<br>0: 99.79%	0.00%
Heart disease	Whether a patient has heart disease	Categorical	1: 1.87%<br>0: 98.13%	0.00%

Lung disease	Whether a patient has lung disease	Categorical	1: 1.45%<br>0: 98.55%	0.00%
Diabetes	Whether a patient has diabetes	Categorical	1: 6.17%<br>0: 93.83%	0.00%
Hypertension	Whether a patient has hypertension	Categorical	1: 13.83%<br>0: 86.17%	0.00%

Note: SD: standard deviation

Table E.10: Descriptive statistics for the Nexoid dataset.

Texas Inpatients

Texas inpatient dataset contains 75 variables. Similar to the Washington state hospital discharge data, in this dataset, we explore the relationship between those demographic and health factors and the length of stay in the Texas hospitals. The involved covariates include age, sex, race, ethnicity, location, weekday, risk mortality, severity, DRG and fees with detailed descriptions in **Table E.11**. According to their length of stay in the hospital, the patients are classified into two groups, and the outcome is assigned a value of 1 if the patient's length of stay is greater than or equal to 3 days and 0 otherwise.

Variable	Description	Type	Mean (SD) or level count (% of total size) or number of categories	Missingness (% of the total size)
Outcome	Whether a patient's length of stay is greater than 3 days	Categorical	1: 59.58%<br>0: 40.42%	0.00%
Age	Patient's age groups	Numeric	0: 11.95%<br>1: 1.66%<br>2: 1.62%<br>3: 1.08%<br>4: 1.39%<br>5: 1.58%<br>6: 1.58%<br>7: 4.84%<br>8: 5.28%	0.00%

			9: 4.94%<br>10: 3.75%<br>11: 3.41%<br>12: 3.93%<br>13: 4.96%<br>14: 5.58%<br>15: 5.95%<br>16: 6.47%<br>17: 6.01%<br>18: 5.78%<br>19: 5.32%<br>20: 3.94%<br>21: 2.40%<br>22: 0.21%<br>23: 2.68%<br>24: 2.86%<br>25: 0.54%<br>26: 0.28%	
Sex	Patient’s gender	Categorical	Female: 56.25%<br>Male: 37.16%	6.58%
Race	Patient’s race	Categorical	1 American Indian/Eskimo/Aleut: 0.77%<br>2 Asian or Pacific Islander: 1.68%<br>3 Black: 12.61%<br>4 White: 61.45%<br>5 Other: 23.35%	0.13%
Ethnicity	Whether a patient is of Hispanic origin	Categorical	1 Hispanic Origin: 28.14%<br>2 Not of Hispanic Origin: 70.45%	1.41%
Location	Patient’s mailing address in Texas and contiguous states	Categorical	AR: 0.48%<br>FC: 0.25%<br>LA: 0.21%<br>NM: 0.57%<br>OK: 0.32%<br>TX: 97.21%<br>XX: 0.02%<br>ZZ: 0.92%	0.02%

Weekday	The day of week a patient is admitted	Categorical	1 Monday: 16.97%<br>2 Tuesday: 17.22%<br>3 Wednesday: 16.38%<br>4 Thursday: 15.89%<br>5 Friday: 14.98%<br>6 Saturday: 9.42%<br>7 Sunday: 9.14%	0.00%
Risk mortality	Risk of mortality score from the All Patient Refined (APR) Diagnosis Related Group (DRG) from the 3M™ APR-DRG Grouper.	Categorical	0 No class specified: 0.10%<br>1 Minor: 60.15%<br>2 Moderate: 20.26%<br>3 Major: 13.26%<br>4 Extreme: 6.22%	0.00%
Severity	Severity of illness score from the All Patient Refined (APR) Diagnosis Related Group (DRG) from the 3M™ APR-DRG Grouper.	Categorical	0 No class specified: 0.10%<br>1 Minor: 35.40%<br>2 Moderate: 33.39%<br>3 Major: 22.76%<br>4 Extreme: 8.35%	0.00%
DRG	All Patient Refined (APR) Diagnosis Related Group (DRG) as assigned by 3M APR-DRG Grouper	Categorical	316	0.00%
Fees	Total non-covered amount of the charge	Numeric	57.51 (1375.47)	0.02%

Note: SD: standard deviation

Table E.11: Descriptive statistics for the Texas inpatient dataset.

Washington State Hospital Discharges

The seventh dataset, Washington State Hospital Discharge dataset, contains over 350 variables. Among these, we model the relationship between those demographic and health factors and the length of stay in the hospital. The covariates were: age, atype, aweekend, died, DRG, primary diagnosis code, and ZIP code. A detailed description of these variables is displayed in **Table E.12**. The outcome of our study categorizes patients into two groups based on their length

of stay. A value of 1 is assigned if the patient's length of stay is greater than or equal to 3 days and 0 otherwise.

Variable	Description	Type	Mean (SD) or 1 count (% of total size) or number of categories	Missingness (% of the total size)
Outcome	Whether a patient's length of stay is greater than 3 days	Categorical	1: 49.07%<br>0: 50.93%	0.00%
Age	Patient's age in years	Numeric	45.58 (28.45)	0.01%
Atype	Admission type	Categorical	1: 34.69%<br>2: 18.03%<br>3: 34.23%<br>4: 12.81%<br>5: 0.23%	0.00%
Aweekend	Whether admission occurs on a weekend	Categorical	1: 19.32%<br>0: 80.68%	0.00%
Died	Whether a patient died during hospitalization	Categorical	1: 1.99%<br>0: 98.01%	0.00%
DRG	Diagnosis-related-group (DRG) in effect on discharge date	Categorical	862	0.00%
DX1	Primary diagnosis	Categorical	5,864	15.14%
ZIP	Patient's ZIP code	Categorical	4,272	0.06%

Note: SD: standard deviation

Table E.12: Descriptive statistics for the hospital Washington dataset.

Washington State Hospital Discharges (2008)

This dataset contains 652,340 inpatient discharge records in 2008 from community hospitals in Washington from State Inpatient Databases that are used to track the trends in healthcare utilization, access, charges, quality and outcomes in the United States. We are interested in examining the relationship between length of stay and demographic and health factors. Specifically, the covariates of interest include age, female, race, admission type, aweekend, DRG, DX1, primary payer, total charges, zip code, chronic conditional indicators, and procedure classes for ICD-10-PCS procedure codes, comorbidity measures for alcohol abuse, depression, hypertension and obesity. The outcome is created by classifying the patients into two groups based on the median of their length of stay. A value of 1 is assigned if the patient's length of stay is greater than or equal to 2 days and 0 otherwise. Detailed statistics of the variables are displayed in **Table E.13**.

Variable	Description	Type	Mean (SD) or level count (% of total size) or number of categories	Missingness (% of the total size)
Outcome	Whether a patient's length of stay is greater than 2 days	Categorical	1: 74.58%<br>0: 25.42%	0.00%
AGE	Patient's age in years	Numeric	45.79 (28.43)	0.01%
FEMALE	Whether a patient's gender is female	Categorical	1 Female: 58.69%<br>0 Male: 41.31%	0.01%
RACE	Patient's race	Categorical	1 White: 23.86%<br>2 Black: 1.18%<br>3 Hispanic: 2.80%<br>4 Asian or Pacific Islander: 1.31%<br>5 Native American: 0.48%<br>6 Other: 0.02%	70.34%
ATYPE	Admission type	Categorical	1 Emergency: 35.59%<br>2 Urgent: 17.80%<br>3 Elective: 33.22%<br>4 Newborn: 12.74%<br>5 Trauma Center: 0.65%	0.00%

AWEEKEND	Whether a patient's admission day is on a weekend	Categorical	1 Admitted Saturday - Sunday: 19.50%<br>0 Admitted Monday - Friday: 80.50%	0.00%
DRG	Diagnosis Related Group	Categorical	746	0.00%
DX1	ICD-9-CM Diagnosis	Categorical	6,149	0.01%
PAY1	Expected primary payer (Medicare, Medicaid, private insurances, etc.)	Categorical	1 Medicare: 31.21%<br>2 Medicaid: 20.03%<br>3 Private insurance: 42.89%<br>4 Self-pay: 2.80%<br>5 No charge: 0.60%<br>6 Other: 2.46%	0.00%
TOTCHG	Total charges	Numeric	26,040.52 (43,943.20)	0.01%
ZIP	Zip code	Categorical	4,191	0.06%
CHRON1	ICD-9-CM Chronic Condition Indicators	Categorical	1 Chronic condition: 35.16%<br>0 Non-chronic condition: 64.82%	0.01%
CHRONB1	Chronic Condition Indicators - body system	Categorical	19	0.01%
PCLASS1	Procedure Classes Refined for ICD-10-PCS procedure codes	Categorical	1 Minor diagnostic: 6.66%<br>2 Minor therapeutic: 24.98%<br>3 Major diagnostic: 0.44%<br>4 Major Therapeutic: 31.07%	36.84%
CM_ALCOH	AHRQ comorbidity measure for ICD-9-CM codes: alcohol abuse	Categorical	1 Comorbidity is present: 3.01%<br>0 Comorbidity is not present: 96.99%	0.00%

CM_DEPRE	AHRQ comorbidity measure for ICD-9-CM codes: depression	Categorical	1 Comorbidity is present: 6.31%<br>0 Comorbidity is not present: 93.69%	0.00%
CM_HTN_C	AHRQ comorbidity measure for ICD-9-CM codes: hypertension (combine uncomplicated and complicated)	Categorical	1 Comorbidity is present: 28.69%<br>0 Comorbidity is not present: 71.31%	0.00%
CM_OBESE	AHRQ comorbidity measure for ICD-9-CM codes: obesity	Categorical	1 Comorbidity is present: 5.55%<br>0 Comorbidity is not present: 94.45%	0.00%

Note: SD: standard deviation

Table E.13: Descriptive statistics for the hospital Washington2008 dataset.

F. Details on the statistical analysis procedure

1. Preparation for statistical testing:
   a. For each dataset, we collected:
      i. AUC scores from the holdout test set
      ii. ICI (Integrated Calibration Index) values from the holdout test set
      iii. These metrics were collected for all model configurations using their optimal n' (determined from validation set)
2. Analysis for Q-1 (Pre-trained vs Non-pre-trained SDG Models):
   a. For each dataset:
      i. Selected the best performing pre-trained SDG model (highest AUC)
      ii. Selected the best performing non-pre-trained SDG model (highest AUC)
      iii. Repeated the selection process for lowest ICI
   b. Conducted two one-tailed paired permutation tests:
      i. Test 1: AUC scores of best pre-trained vs best non-pre-trained SDG models
      ii. Test 2: ICI scores of best pre-trained vs best non-pre-trained SDG models
   c. Null hypothesis: Pre-trained SDG models do not perform better than non-pre-trained SDG models
   d. Alternative hypothesis: Pre-trained SDG models perform better than non-pre-trained SDG models
3. Analysis for Q-2 (Impact of Augmentation):
   a. For each classifier (LGBM and TabPFN):
      i. Collected performance metrics with no augmentation
      ii. Collected performance metrics with best augmentation strategy (from Q-1)
   b. Conducted four one-tailed paired permutation tests:
      i. Test 1: AUC scores for LGBM (augmented vs non-augmented)
      ii. Test 2: ICI scores for LGBM (augmented vs non-augmented)
      iii. Test 3: AUC scores for TabPFN (augmented vs non-augmented)

        iv. Test 4: ICI scores for TabPFN (augmented vs non-augmented)
    c. Null hypothesis: Augmentation does not improve model performance
    d. Alternative hypothesis: Augmentation improves model performance

4. Analysis for Q-3 (Best Overall Classifier):
    a. Used each classifier's best configuration:
        i. LGBM with its optimal augmentation strategy
        ii. LLMs without augmentation
        iii. TabPFN with its optimal augmentation strategy
    b. Conducted six one-tailed paired permutation tests for each data regime (n0=50 and n0=350):
        i. Test 1: AUC scores (LGBM vs LLM)
        ii. Test 2: AUC scores (TabPFN vs LGBM)
        iii. Test 3: AUC scores (TabPFN vs LLM)
        iv. Test 4: ICI scores (LGBM vs LLM)
        v. Test 5: ICI scores (TabPFN vs LGBM)
        vi. Test 6: ICI scores (TabPFN vs LLM)
    c. For each test:
        i. Null hypothesis: Model A does not perform better than Model B
        ii. Alternative hypothesis: Model A performs better than Model B

5. Documentation of Results:
    a. Recorded p-values for all statistical tests
    b. Applied appropriate multiple testing corrections if necessary
    c. Created summary tables showing:
        i. Test statistics
        ii. P-values
        iii. Effect sizes

d. Highlighted statistically significant results ($p < 0.05$)

This detailed statistical analysis plan ensured systematic evaluation of our research questions while maintaining statistical rigor and transparency in our methodology.

G. Fidelity check

The following table shows the mean Hellinger distance between the real dataset and synthetic for each generative method.

	Distilgpt2	Llama 1B	Llama 8B	Sequential trees	Bayesian network	CTGAN	TVAE
Born	0.163	0.171	0.167	0.240	0.287	0.272	0.267
BSA	0.172	0.123	0.136	0.091	0.105	0.139	0.155
California	0.280	0.315	0.163	0.378	0.344	0.372	0.398
CCHS	0.238	0.155	0.213	0.074	0.088	0.123	0.143
Covid	0.256	0.178	0.272	0.144	0.180	0.391	0.281
FAERS	0.302	0.149	0.339	0.210	0.226	0.399	0.359
florida	0.191	0.137	0.202	0.184	0.265	0.310	0.335
MIMIC	0.319	0.223	0.304	0.053	0.082	0.151	0.184
Newyork	0.305	0.179	0.239	0.149	0.157	0.191	0.224
Nexoid	0.165	0.141	0.164	0.050	0.034	0.075	0.068
Texas	0.207	0.182	0.201	0.042	0.043	0.086	0.093
Washington	0.160	0.155	0.205	0.152	0.129	0.289	0.202
Washington2008	0.213	0.125	0.173	0.085	0.096	0.123	0.130

H. References

(1) Pilgram, L.; Dankar, F. K.; Drechsler, J.; Elliot, M.; Domingo-Ferrer, J.; Francis, P.; Kantarcioglu, M.; Kong, L.; Malin, B.; Muralidhar, K.; Myles, P.; Prasser, F.; Raisaro, J. L.; Yan, C.; Emam, K. E. A Consensus Privacy Metrics Framework for Synthetic Data. *Patterns* **2025**, *0* (0). https://doi.org/10.1016/j.patter.2025.101320.

(2) Azizi, Z.; Lindner, S.; Shiba, Y.; Raparelli, V.; Norris, C. M.; Kublickiene, K.; Herrero, M. T.; Kautzky-Willer, A.; Klimek, P.; Gisinger, T.; Pilote, L.; El Emam, K. A Comparison of Synthetic Data Generation and Federated Analysis for Enabling International Evaluations of Cardiovascular Health. *Sci. Rep.* **2023**, *13* (1), 11540. https://doi.org/10.1038/s41598-023-38457-3.

(3) Lloyd-Jones, D. M.; Hong, Y.; Labarthe, D.; Mozaffarian, D.; Appel, L. J.; Van Horn, L.; Greenlund, K.; Daniels, S.; Nichol, G.; Tomaselli, G. F.; Arnett, D. K.; Fonarow, G. C.; Ho, P. M.; Lauer, M. S.; Masoudi, F. A.; Robertson, R. M.; Roger, V.; Schwamm, L. H.; Sorlie, P.; Yancy, C. W.; Rosamond, W. D. Defining and Setting National Goals for Cardiovascular

Health Promotion and Disease Reduction. *Circulation* **2010**, *121* (4), 586–613. https://doi.org/10.1161/CIRCULATIONAHA.109.192703.

(4) Maclagan, L. C.; Park, J.; Sanmartin, C.; Mathur, K. R.; Roth, D.; Manuel, D. G.; Gershon, A.; Booth, G. L.; Bhatia, S.; Atzema, C. L.; Tu, J. V. The CANHEART Health Index: A Tool for Monitoring the Cardiovascular Health of the Canadian Population. *CMAJ* **2014**, *186* (3), 180–187. https://doi.org/10.1503/cmaj.131358.

(5) Johnson, A. E. W.; Pollard, T. J.; Shen, L.; Lehman, L. H.; Feng, M.; Ghassemi, M.; Moody, B.; Szolovits, P.; Anthony Celi, L.; Mark, R. G. MIMIC-III, a Freely Accessible Critical Care Database. *Sci. Data* **2016**, *3* (1), 160035. https://doi.org/10.1038/sdata.2016.35.

(6) Goldberger, A. L.; Amaral, L. A. N.; Glass, L.; Hausdorff, J. M.; Ivanov, P. Ch.; Mark, R. G.; Mietus, J. E.; Moody, G. B.; Peng, C.-K.; Stanley, H. E. PhysioBank, PhysioToolkit, and PhysioNet: Components of a New Research Resource for Complex Physiologic Signals. *Circulation* **2000**, *101* (23). https://doi.org/10.1161/01.CIR.101.23.e215.

(7) Johnson, A.; Pollard, T.; Mark, R. MIMIC-III Clinical Database (Version 1.4), 2016. https://doi.org/10.13026/C2XW26.
